# Peroxynitrite-mediated tyrosine nitration modulates β-1,3-glucanase activity and potato defense against *Phytophthora infestans*

**DOI:** 10.3389/fpls.2026.1796014

**Published:** 2026-04-17

**Authors:** Karolina Izbiańska-Jankowska

**Affiliations:** Department of Plant Ecophysiology, Institute of Experimental Biology, Faculty of Biology, Adam Mickiewicz University, Poznan, Poland

**Keywords:** peroxynitrite, *Phytophthora infestans*, potato, tyrosine nitration, β-1,3-glucanase

## Abstract

Protein tyrosine nitration is a well-documented peroxynitrite (ONOO^–^)- mediated post-translational modification (PTM) in biological systems; however, its relevance in plant immune response remains poorly understood. In this study, a potato–avr/vr *Phytophthora infestans* system was used as a model to investigate the functional consequences of protein tyrosine nitration. This work shows that only the potato-avr *P. infestans* interaction is accompanied by an early and transient accumulation of nitrated proteins, observed within the first 6 hpi. In turn, the vr *P. infestans* pathosystem exhibited a delayed and prolonged accumulation of nitrated protein with maximum levels coinciding with tissue colonization. Two-dimensional gel electrophoresis coupled with immunodetection identified β-1,3-glucanase (PR-2), a key enzyme in plant resistance, as one of the main targets of tyrosine nitration. Functional studies using recombinant β-1,3-glucanase demonstrated that peroxynitrite-mediated nitration directly inhibits enzymatic activity. *In silico* structural analysis indicated that Tyr59 in the catalytic pocket emerges as a key regulatory site. Collectively, these results suggest that protein tyrosine nitration represents a dynamic and selective regulatory mechanism in potato immunity.

## Introduction

One of the first reactions of plants to pathogen attack is the activation of signal transduction pathways, leading, e.g., to changes in gene expression and the activity of specific pathogenesis-related proteins (PRs) involved in preventing pathogen spread (Trapet et al., 2015; Floryszak-Wieczorek and Arasimowicz-Jelonek, 2016). As demonstrated, in this coordinated transfer of information about the emerging threat, endogenous signaling molecules, such as nitric oxide (NO), play a particularly significant role. What is more, recent advances in the understanding of plant defense signaling pathways have proven that NO is a key component engaged in pathogen recognition and in the activation of local and systemic defense mechanisms (e.g., Bellin et al., 2013; Bigeard et al., 2015; Floryszak-Wieczorek and Arasimowicz-Jelonek, 2016; Khan et al., 2023). Importantly, the burst of NO, occurring rapidly after pathogen recognition, may lead in synergy with reactive oxygen species (ROS) to the formation of other reactive nitrogen species (RNS), which in turn substantially alter the cellular redox balance and trigger further metabolic defense responses (e.g., Floryszak-Wieczorek and Arasimowicz-Jelonek, 2016; Bleau and Spoel, 2021).

Peroxynitrite (ONOO^–^) is one of the best-known NO-derived species in biological systems. This powerful oxidant and nitrating agent is formed in an extremely rapid, diffusion-controlled reaction between NO and superoxide (O_2_^•−^) (Bartesaghi and Radi, 2018; Möller and Denicola, 2024). As a highly reactive and short-lived molecule, peroxynitrite was originally recognized as a toxic compound contributing to oxidative and nitrosative stress within cells (Vandelle and Delledonne, 2011; Corpas et al., 2013b). However, an increasing number of studies have reported that ONOO^–^ cannot be considered only as a mediator of cellular dysfunction, but also behaves as a potent modulator of the redox regulation in multiple cell signaling pathways, mainly through the post-translational modification (PTM) of proteins by tyrosine (Tyr) nitration (Arasimowicz-Jelonek and Floryszak-Wieczorek, 2011; Gzyl et al., 2016; León, 2022).

Protein Tyr nitration is a covalent modification that introduces a nitro group (–NO_2_) into one of the two equivalent *ortho*-carbons of the aromatic ring of Tyr residues, leading to the formation of 3-nitrotyrosine (Kolbert et al., 2017). This biochemical event converts the Tyr molecule into a negatively charged, hydrophilic nitrotyrosine moiety. It causes a marked shift in the local pKa of the hydroxyl group from 10.07 in tyrosine to 7.50 in nitrotyrosine (Begara-Morales et al., 2013; Ferrer-Sueta et al., 2018). This is thought to affect the activity, stability, or intracellular location of proteins, thereby potentially altering their functions, protein-protein interactions, and ultimately cell signaling (Kolbert et al., 2017). It has been experimentally confirmed that nitration of critical Tyr residues typically results in a loss of function (Kolbert et al., 2017) or no change in protein function (Begara-Morales et al., 2013). However, it should be emphasized that, in animal systems, increasing evidence suggests that tyrosine nitration is reversible and, consequently, has a signaling function (Sabadashka et al., 2021). Importantly, tyrosine nitration is a selective process with a well-defined target: not all tyrosine residues in a protein are nitrated, nor are all proteins targets for nitration (Kolbert et al., 2017). Experiments performed on animal organisms have shown that only 1 to 5 out of 10,000 Tyr residues in the whole cell/tissues undergo this post-translational modification (Bartesaghi and Radi, 2018; Ferrer-Sueta et al., 2018).

In plants, the role of tyrosine nitration in pathogen interactions remains poorly understood. To date, only two published reports have identified potential protein targets in plants challenged with a pathogen using mass spectrometry (Cecconi et al., 2009; Arasimowicz-Jelonek et al., 2016). What is more, the functional effect of this modification has been confirmed only for 22 proteins, wherein none of them was specifically related to the plant-pathogen interaction (Alvarez et al., 2011; Galetskiy et al., 2011; Lozano-Juste et al., 2011; Begara-Morales et al., 2013, Begara-Morales et al., 2014, Begara-Morales et al., 2015, Begara-Morales et al., 2019; Corpas et al., 2013a; Castillo et al., 2015; Holzmeister et al., 2015; Sainz et al., 2015; Takahashi et al., 2015, Takahashi et al., 2016; Costa-Broseta et al., 2020, Costa-Broseta et al., 2021; Méndez et al., 2020) (**Supplementary Table 1**). Nevertheless, some representatives belonging to the family of pathogenesis-related proteins (PRs) have been identified as potential candidates for Tyr nitration (Lozano-Juste et al., 2011; Tanou et al., 2012; Arasimowicz-Jelonek et al., 2016; Takahashi et al., 2016). Although little is known about the specific impact of NO-related PTMs on the function/activity of PR proteins, it should be stressed that in plants nitration has been shown to mainly provoke a loss of protein function (e.g., Lozano-Juste et al., 2011; Corpas et al., 2013a; Begara-Morales et al., 2014; Holzmeister et al., 2015). Thus, pathogenesis-related Tyr nitration of proteins could negatively regulate plant defense. However, to the best of the authors’ knowledge, no information is available on the role of ONOO^–^ in regulating the activity of the important class of defense response proteins.

The previous studies have shown that ONOO^–^ formation in potato (*Solanum tuberosum* L.) leaves is closely associated with both basal resistance and hypersensitive cell death in resistant potato genotypes (Arasimowicz-Jelonek et al., 2016; Izbiańska et al., 2018). Since β-1,3-glucanases are well-known components of plant innate immunity (e.g., dos Santos and Franco, 2023), the present work is dedicated to characterizing the functional role of protein Tyr nitration in plant responses to pathogen attack and resistance. The study examined the ONOO^–^ effect on the post-translational regulation of β-1,3-glucanase to assess whether selective nitration accompanies effective defense activation or contributes to disease development. The experiments were conducted on the leaves of two potato genotypes that differ radically in their resistance to *Phytophthora infestans*. The use of a highly resistant and a susceptible cultivar to *P. infestans* created a useful background for a comparative study. Recombinant β-1,3-glucanase was used to assess the direct effect of nitration on enzyme activity, while bioinformatics tools were applied to identify potential nitration sites. This study provides new insights into the role of protein nitration in potato defense responses and reveals cultivar-specific differences in ONOO^–^-dependent regulation of the antifungal activity of key defense proteins.

## Materials and methods

### Plant material and growth conditions

The experiments were conducted on leaves of two sterile potato cultivars (*Solanum tuberosum* L.) – cv. ‘Bintje’ (lacking R genes), which is highly susceptible to isolate 1.3.4.7.10.11. *Phytophthora infestans*, and cv. ‘Bzura’ [carrying the R1 gene (Gebhardt et al., 2004) and the R2-like gene located in chromosome IV (Plich et al., 2015)] – highly resistant to 1.3.4.7.10.11. *P. infestans*. Plants of both cultivars derived from *in vitro* tissue culture were transferred to soil and grown in a phytochamber with 16 hours of light (180 μmol m^-2^ s^-1^) at 18 ± 2 °C and 40% humidity until they reached the ten-leaf stage. Both potato cultivars were obtained from the Potato Gene Bank (Plant Breeding and Acclimatization Institute, National Research Institute Branch in Bonin, Poland).

### Pathogen culture

*Phytophthora infestans* (Mont.) de Bary – isolate 1.3.4.7.10.11, virulent to ‘Bintje’ and avirulent (causing hypersensitive reaction) to ‘Bzura’ was kindly supplied by the Plant Breeding and Acclimatization Institute, Research Division at Młochów, Poland. The oomycete was grown on a cereal-potato medium supplemented with dextrose (Arasimowicz-Jelonek et al., 2016).

### Method of inoculation and sample collection

For *P. infestans* inoculation, the abaxial surface of detached leaves from both potato cultivars was sprayed with a zoospore suspension prepared in sterile water (concentration 2.0 × 10^5^ per ml) according to the protocol described by Arasimowicz-Jelonek et al. (2016). The plants were kept at 100% humidity in a growth chamber. The material for further analysis was taken up to 96 h post-inoculation (hpi). For RNA and protein extraction, the leaves were frozen immediately in liquid nitrogen and stored at -80 °C until use. Three replicates of control (mock-inoculated) and *P. infestans*-inoculated leaves of both cultivars were harvested at each time point. Three leaves per treatment were maintained for 7 days post-inoculation to monitor disease progression and confirm the effectiveness of the inoculation.

Additionally, RT-qPCR was used to quantify infection levels in plant tissues. The transcript levels of the *P. infestans* translation elongation factor 1*α* (*PiTef1*) gene, which is highly and constitutively expressed throughout all developmental stages of the pathogen (Orłowska et al., 2012), were analyzed in leaves of both potato genotypes at each time point after inoculation. For all samples, *PiTef1* expression was normalized against the potato elongation factor 1*α* (*EF-1α*) reference gene (Orłowska et al., 2012).

### Peroxynitrite donor and scavenger treatment

To evaluate the impact of peroxynitrite (ONOO^–^) -mediated tyrosine nitration on β-1,3-glucanase (PR-2) expression and enzymatic activity, potato leaves (*in vivo*), and recombinant PR-2 protein (*in vitro*) were incubated for 3 and 1 h, respectively, with increasing concentrations (0.05–2 mM) of the ONOO^–^ donor, 3-morpholinosydnonimine (SIN-1; Calbiochem). SIN-1 gradually decomposes to yield equimolar amounts of NO and O_2_˙¯, which subsequently react to form ONOO^–^. Following incubation, the *in vitro* samples were passed through a NAP-10 column to remove SIN-1 and prevent interference with the activity assay. To assess the effect of endogenous ONOO^–^ on protein Tyr nitration, a peroxynitrite scavenger (0.05 mM ebselen, Sigma) was used. The control samples were treated with sterile water. After 3 h of incubation, the leaves were gently dried and inoculated as described above.

### Protein 3-nitrotyrosine assay

Leaves (0.25 g) were ground to a fine powder in liquid nitrogen (N_2_) and suspended in a ratio of 1:3 (w/v) in 50 mM Tris-HCl buffer (pH 7.6) supplemented with 0.6% PVPP, 1 mM PMSF, and plant protease inhibitor cocktail (Sigma). Crude extracts were centrifuged at 10,000 *g* for 15 min at 4 °C. The concentration of supernatant proteins was quantified using the Bradford assay (Bradford, 1976), with bovine serum albumin (BSA) as a standard. 3-nitrotyrosine (3-NT) levels were quantified using the OxiSelect™ Nitrotyrosine ELISA Kit (Cell Biolabs) according to the manufacturer’s instructions. Optical density was measured at 450 nm using an iMark microplate reader (Bio-Rad). The 3-NT content in protein samples was determined from a prepared standard curve. Each sample was analyzed in triplicate, and the data represent the means of three biological replicates ± SD.

### Nitroproteome analysis

Protein extraction for 2D electrophoresis was performed according to Hurkman and Tanaka (1986) with slight modifications. Briefly, 0.2 g of powdered leaves was homogenized with 500 µL of extraction buffer (0.7 M sucrose, 0.5 M TRIS, 30 mM HCl, 50 mM EDTA, 2% DTT, and 0.1 M KCl). An equal volume of phenol was then added, vortexed, and centrifuged at 20,000 *g* for 15 min. An upper phenol phase was transferred to new tubes, and 500 µL of extraction buffer was added. After vortexing and centrifuging under the same conditions, proteins from the phenol phase were precipitated by adding five volumes of cold 0.1 M ammonium acetate in methanol and incubating overnight at −20 °C. After centrifugation (9000 g, 0 °C, 30 min), the precipitate was washed once with cold ammonium acetate in methanol and twice with cold acetone. The proteins were then solubilized using DeStreak Rehydration Solution (GE Healthcare), supplemented with 20 mM DTT and 0.2% IPG buffer (pH 4-7) (GE Healthcare). Protein concentration in the final samples was determined using a commercial 2-D Quant Kit (GE Healthcare) according to the manufacturer’s instructions. Approximately 100 μg of protein was loaded onto 7 cm IPG strips with a 4–7 pH gradient (Bio-Rad). The strips were rehydrated overnight and used for isoelectrofocusing (IEF) using a Protean IEF cell system (Bio-Rad). The run was carried out in the following order: (i) 300 V (1 h), (ii) 3500 V (1.5 h), and (iii) 3500 V (total 20,000 Vh). For the SDS-PAGE, the strips were equilibrated 2 times for 15 min in an equilibration buffer (50 mM Tris–HCl, pH 8.8; 6 M urea, 30% glycerol, 2% SDS, 0.002% Bromophenol blue), first containing 65 mM DTT, followed by an equilibration buffer with 135 mM iodoacetamide. For separation in the second dimension, the strips were applied to 13% SDS PAGE gels and run in a Mini-PROTEAN Tetra Cell (Bio-Rad) at a constant current (20 mA per gel) with a Prestained Protein Ladder (Thermo Scientific). For Western blot analyses, proteins were transferred to PVDF membranes. After transfer, membranes were used for cross-reactivity assays with rabbit polyclonal antibodies against nitrotyrosine (Life Technologies, 1:1000 dilutions). Specificity of the anti-nitrotyrosine antibodies was verified by reducing nitrotyrosine to aminotyrosine using sodium dithionite, as described by Izbiańska et al. (2019). For immunodetection, the goat anti-rabbit antibody conjugated to horseradish peroxidase (Agrisera) and Clarity Max Western ECL Substrate (Bio-Rad) were used. Spot intensities were quantified using a ChemiDoc MP Imaging System (Bio-Rad) equipped with a high-resolution camera, and PDQuest 2-D Analysis Software (Bio-Rad). To identify nitrotyrosine-containing proteins by Mass Spectrometry procedure, blots were incubated with a rabbit primary polyclonal anti-nitrotyrosine antibody (Life Technologies, 1:1000 dilutions) and an anti-rabbit secondary antibody conjugated with alkaline phosphatase (Sigma). Detection was performed using SIGMAFAST™ (BCIP^®^/NBT, Sigma). Positive spots were then identified using a MALDI-TOF mass spectrometer according to procedures recommended by the Laboratory of Mass Spectrometry, IBB PAS (Warsaw, Poland).

### Mass spectrometry and protein identification

Protein identification was performed using liquid chromatography-tandem mass spectrometry (LC-MS/MS) at the Laboratory of Mass Spectrometry, Institute of Biochemistry and Biophysics, Polish Academy of Sciences (Warsaw, Poland). Protein spots were manually excised from the PVDF membranes under a binocular microscope using a sterile, disposable razor blade and subjected to the standard trypsin digestion procedure, as described earlier (Arasimowicz-Jelonek et al., 2016; Izbiańska et al., 2019). Raw data were pre-processed with the Mascot Distiller software (v. 2.4.2.0; Matrix Science), then obtained peptide masses and fragmentation spectra were matched to the National Center Biotechnology Information (NCBI) non-redundant database (37,425,594 sequences; 13,257,553,858 residues), with a *Viridiplantae* filter (1,760,563 sequences) using the mascot search engine (Mascot Daemon v. 2.4.0, Mascot Server v. 2.4.1; Matrix Science). The following parameters were used for database searches: enzyme specificity was set to semiTrypsin, peptide mass tolerance to 20 ppm, and fragment mass tolerance to 0.1 Da. The protein mass was left unrestricted, and mass values were monoisotopic, with one allowed missed cleavage. Alkylation of cysteine by carbamidomethylation was fixed, and oxidation of methionine and carboxymethylation on lysine were set as variable modifications. Protein identification was performed using the Mascot search engine, with the probability-based algorithm. The expected value threshold of 0.05 was used for analysis, meaning that all peptide identifications had a probability of less than 1 in 20 of being random matches.

### Determination of β-1,3-glucanase protein accumulation

Leaves (0.25 g) were ground in liquid N_2_ to a fine powder and then suspended in a ratio of 1:3 (w/v) in 50 mM sodium acetate buffer (pH 5.0) supplemented with 4 mM DTT, 0.6% PVPP, 1 mM PMSF, and plant protease inhibitor cocktail (Sigma). The crude extracts were centrifuged at 10,000 *g* for 15 min at 4 °C. The concentration of supernatant proteins was then determined using the Bradford assay (Bradford, 1976), with bovine serum albumin (BSA) as a standard. The protein samples (50 μg each) were mixed with Laemmli buffer, denatured at 70 °C for 10 min, and separated on an SDS-PAGE gel. The gels were either stained with Coomassie Brilliant Blue G-250 or electroblotted onto PVDF membranes. After transfer, the membranes were blocked (3% BSA) and incubated with polyclonal antibodies against β-1,3-glucanase (Agrisera) at a 1:1000 dilution. For immunodetection, a goat anti-rabbit antibody conjugated to horseradish peroxidase (Agrisera) and Clarity Max Western ECL Substrate (Bio-Rad) were used. Band intensities were quantified using a ChemiDoc MP Imaging System (Bio-Rad) coupled with a high-resolution camera.

### Gene expression measurement

For RNA extraction, leaves (0.15 g) were ground to a fine powder in liquid nitrogen, and total RNA was isolated using TriReagent (Sigma) according to the manufacturer’s instructions. To remove genomic DNA contamination, the RNA samples were treated with DNase I using the Deoxyribonuclease Kit (Sigma). For the reverse transcription, 1 μg of RNA was processed using a Reverse Transcription Kit (Thermo Scientific) according to the manufacturer’s instructions. Real-time PCR was performed on a Rotor-Gene 6000 Thermocycler (Qiagen). All primers (**Supplementary Table 2**) were designed with Primer3 software based on *Solanum tuberosum* cDNA sequences available in the NCBI (GenBank) database. The reaction specificity and *Cq* values for individual samples were determined using the Real-time PCR Miner algorithm (Zhao and Fernald, 2005). The data were normalized to a reference gene encoding potato elongation factor 1*α* (*EF-1α*). Relative gene expression was calculated using the efficiency-corrected models described by Pfaffl (2001).

### Determination of β-1,3-glucanase activity

The β-1,3-glucanse activity was determined according to the procedure proposed by Abeles and Forrence (1970) using a colorimetric assay with laminarin as a substrate. The β-1,3-glucanase activity was determined by measuring the release of reducing sugars (glucose equivalents) and expressed as nmol glucose mg^−1^ protein min^−1^.

To study β-1,3-glucanase active isoforms, zymographic analysis was performed. Protein extracts were separated by SDS-PAGE on 12.5% (w/v) polyacrylamide gels supplemented with 0.01% (w/v) laminarin. After electrophoresis, proteins were renatured by incubating the gels overnight in 50 mM sodium acetate buffer (pH 5.0) containing 1% (v/v) Triton X-100. Enzymatic activity was subsequently detected as described by Pan et al. (1991). The gels were incubated in 50 mM sodium acetate buffer (pH 5.0) for 1 h at 37 °C, fixed in a methanol:water: acetic acid mixture (5:5:2, v/v/v) for 5 min, and rinsed with water. Activity bands were visualized by boiling the gels in 200 mL of 1.0 M NaOH containing 0.15% (w/v) 2,3,5-triphenyltetrazolium chloride (Sigma). Red bands indicating β-1,3-glucanase activity appeared within 5–10 min.

### Recombinant β-1,3-glucanase source and functional characterization

Recombinant *Solanum tuberosum* β-1,3-glucanase (PR-2; UniProt ID: P52401) corresponding to the sequence identified in the nitroproteome analysis was obtained from Cusabio (Houston, USA). The enzyme was produced in an *Escherichia coli* expression system, with a purity of at least 85% as specified by the manufacturer. Protein identity and purity were independently verified in-house *via* SDS-PAGE and Western blot analysis. Before *in vitro* nitration experiments, the basal enzymatic activity of the recombinant PR-2 was confirmed using the laminarin-based colorimetric assay described by Abeles and Forrence (1970).

### Protein modeling and functional analysis

The tertiary structure of β-1,3-glucanase was generated by homology modeling using the SWISS-MODEL server (Arnold et al., 2006). The β-1,3-glucanase from *Hevea brasiliensis* (PDB ID: 4HPG, chain A) was selected as a template due to its higher sequence similarity, lower predicted RMSD, and broader structural coverage compared to available *Solanum tuberosum* sequences. Model quality was evaluated through ANOLEA (Melo and Feytmans, 1998), Verify3D (Eisenberg et al., 1997), and PROCHECK (Laskowski et al., 1993). Three-dimensional structures were visualized and analyzed with UCSF Chimera. Distances between residues (in Å) and hydrogen bonding networks were determined using Chimera’s default geometric parameters. Multiple sequence alignments were performed using Clustal Omega (Madeira et al., 2024). Evolutionary conservation of amino acid residues in potato β-1,3-glucanase (UniProt ID: P52401) was assessed using the ConSurf server (Ashkenazy et al., 2016), with conservation grades ranging from 1 (variable) to 9 (highly conserved). The active site was assigned as UniProt annotations, with E120 acting as the catalytic acid and E265 as the nucleophile. Surface active pocket analysis was conducted using the CASTp 3.0 server with a 1.4 Å probe radius (Tian et al., 2018). Tyrosine residues located within a 5 Å radius of the catalytic site were selected for further analysis as potential nitration targets. The secondary structure of β-1,3-glucanase was determined using the STRIDE algorithm (Frishman and Argos, 1995) based on the generated 3D model.

### Statistical analysis

All results are based on at least three biological replicates derived from three independent experiments. Statistical significance was evaluated using one-way analysis of variance (ANOVA), followed by Dunnett’s *post hoc* test for multiple comparisons (*α* = 0.05). Differences in protein spot intensities were determined using ANOVA followed by a Student’s t-test. In all analyses, a *P* < 0.05 was considered statistically significant.

## Results

### Genotype-specific dynamics of peroxynitrite-mediated protein tyrosine nitration during potato immune response

**Figure 1:**
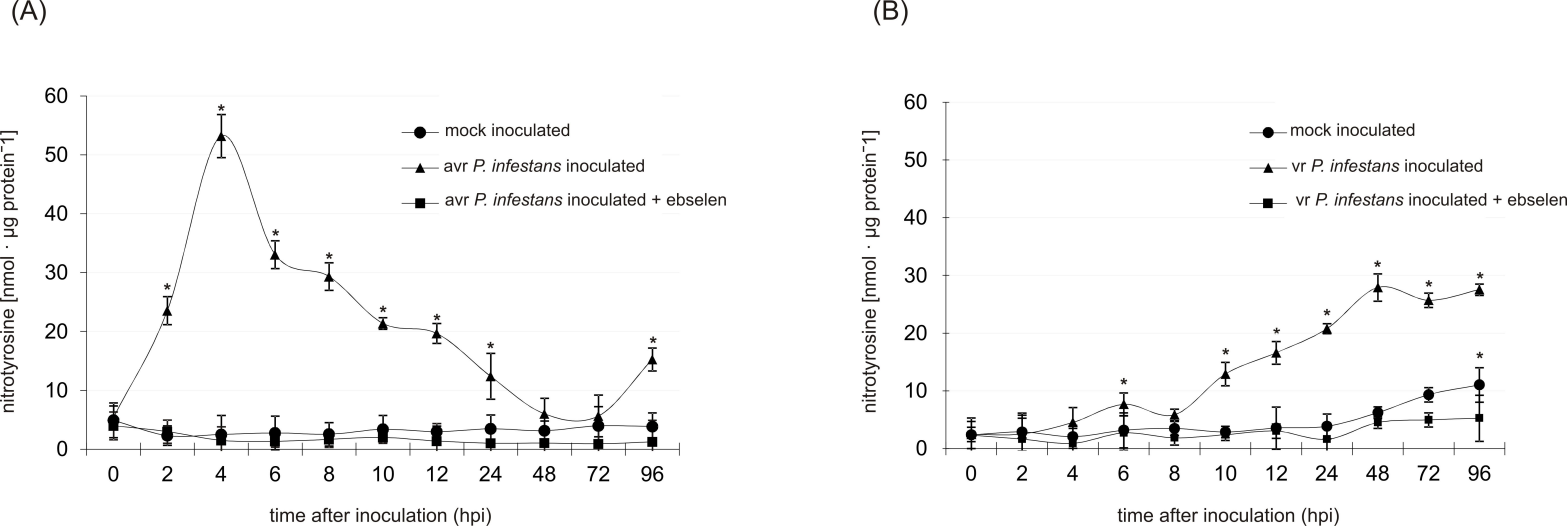
Quantification of protein tyrosine nitration in leaves of resistant **(A)** and susceptible **(B)** potato inoculated with *P. infestans*. Analyses were performed at 0, 2, 4, 6, 8, 10, 12, 24, 48, 72, and 96 h after challenge inoculation. Values represent the mean ± SD of at least three independent experiments (n = 3). Asterisks indicate values that differ significantly from mock-inoculated (control) potato leaves at *P < 0.05.

Using immunoassay and pharmacological approaches, Izbiańska et al. (2018) previously reported that ONOO^–^ can induce tyrosine nitration in cells of both potato genotypes. Furthermore, they also found that *P. infestans* challenge provoked an early and transient accumulation of nitrated proteins only in the resistant potato genotype (Izbiańska et al., 2018). In the present work, the formation of nitrotyrosine in potato leaves was closely monitored up to 96 hours post-inoculation (hpi). As expected, an early and significant rise in the level of nitrated protein was observed only in the potato–avr *P. infestans* interaction, starting from the 2nd hpi (**Figure 1A**). The highest, ca. 50-fold, increase in the total pool of nitrotyrosine-containing proteins was detected at 4 hpi (**Figure 1A**). Then, it gradually decreased and reached the amount noted in mock-inoculated leaves at 48 hpi. However, a substantial, ca. 3-fold increase in the nitrated protein was also observed at 96 hpi (**Figure 1A**). In contrast, the potato-*vr. P. infestans* pathosystem revealed time-delayed accumulation of nitred proteins, starting from 12 hpi (**Figure 1B**). The highest (ca. 7-fold increase) level of nitrated protein was found at 48 hpi (**Figure 1B**). Notably, significantly reduced levels of protein undergoing tyrosine nitration were detected in potato leaves of both cultivars pretreated with ebselen, confirming that pathogen-induced increase in nitrotyrosine accumulation was caused by endogenous ONOO^–^ (**Figures 1A, B**).

### Tyrosine nitration patterns differ between potato genotypes upon pathogen challenge

**Figure 2:**
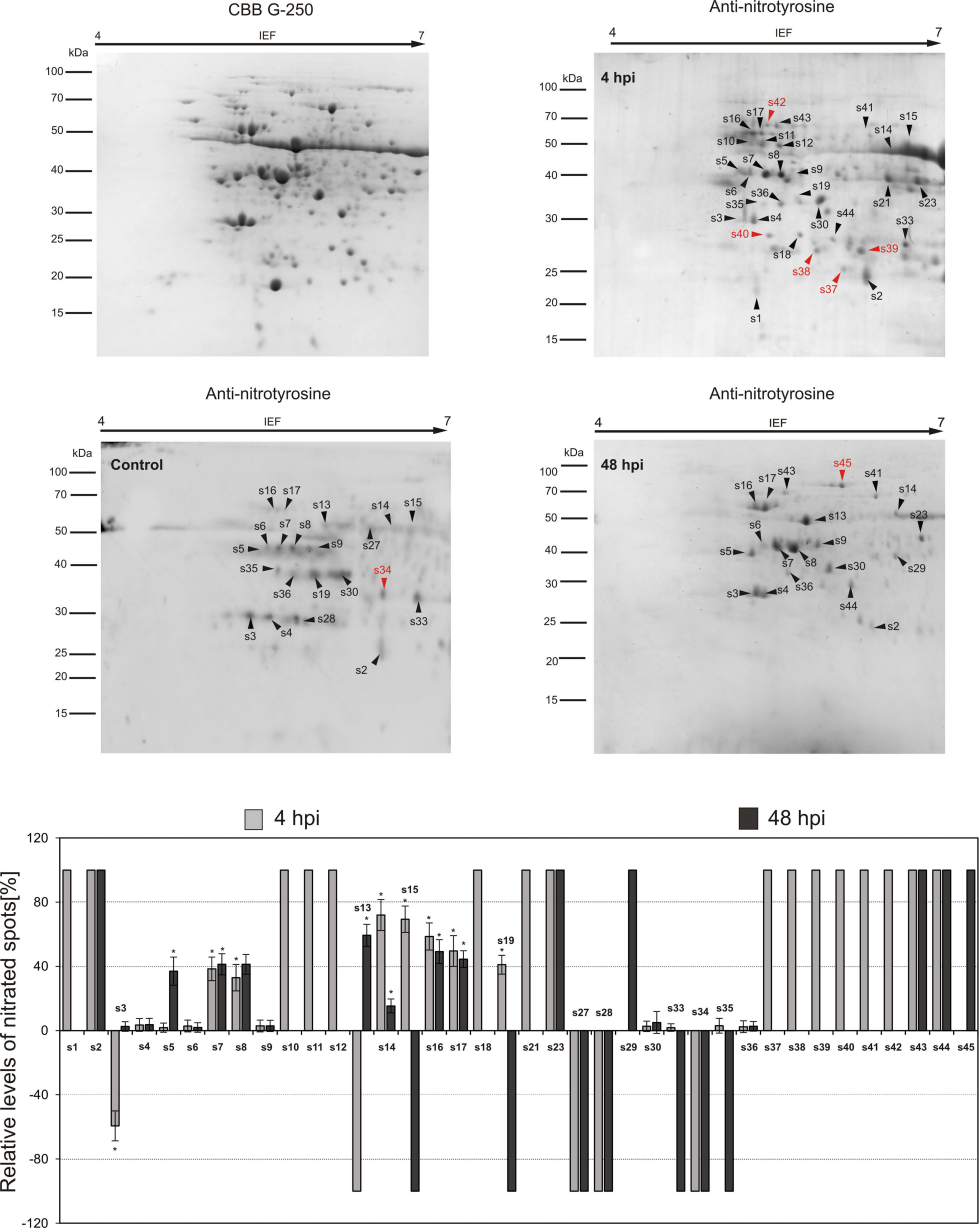
Tyrosine nitration pattern in potato leaves challenge-inoculated with *Phytophthora infestans* at 4 and 48 h post-inoculation (hpi) in the resistant genotype. Representative 2D electrophoresis (pH 4–7 for the first dimension) of potato-avr *P. infestans* stained with CBB G-250 and representative immunoblots probed with a polyclonal antibody against nitrotyrosine diluted at 1:1000. Molecular-mass standards (kDa) are indicated on the left. Arrowheads indicate all the immunoreactive spots, the symbols (s1–s45) refer to the proteins listed in **Table 1**. The quantitative results for protein Tyr nitration were calculated using PDQuest 2-D Analysis Software (Bio-Rad), and the data were presented relative to the control sample, with the average set to 0 (control – mock-inoculated genotype). Asterisks indicate values that differ significantly from the control at *p < 0.05.

**Figure 3:**
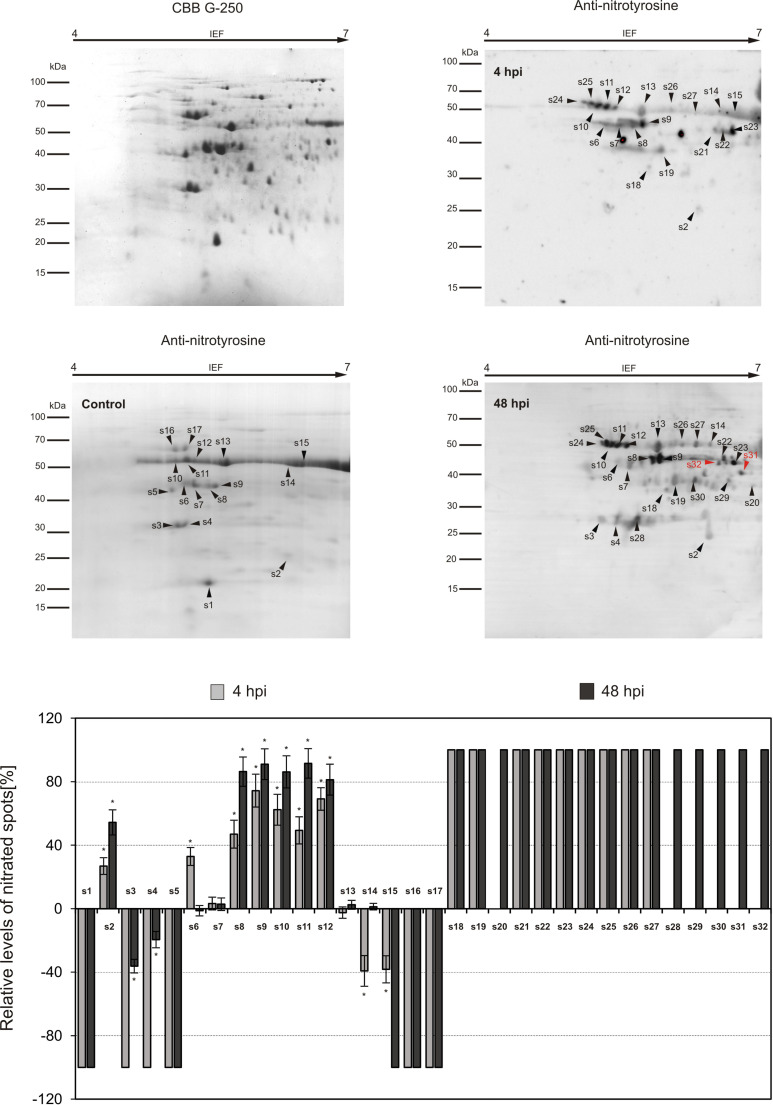
Tyrosine nitration pattern in potato leaves challenge-inoculated with *Phytophthora infestans* at 4 and 48 h post-inoculation (hpi) in the susceptible genotype. Representative 2D electrophoresis (pH 4–7 for the first dimension) of potato-vr *P. infestans* stained with CBB G-250 and representative immunoblots probed with a polyclonal antibody against nitrotyrosine diluted at 1:1000. Molecular-mass standards (kDa) are indicated on the left. Arrowheads indicate all the immunoreactive spots, the symbols (s1–s32) refer to the proteins listed in **Table 1**. The quantitative results for protein Tyr nitration were calculated using PDQuest 2-D Analysis Software (Bio-Rad), and the data were presented relative to the control sample, with the average set to 0 (control – mock-inoculated genotype). Asterisks indicate values that differ significantly from the control at *p < 0.05.

To explore the functional role of protein Tyr nitration in plant immunity, two critical time points (4 and 48 hpi) representing the highest and lowest nitration levels in each potato cultivar were selected for nitroproteome analysis (**Figures 1A, B**). A total of 45 immunopositive spots were detected in potato genotypes. Among these, 38 spots were identified in the potato-avr *P. infestans* interaction (**Figure 2**), while the vr-*P. infestans* pathosystem revealed the 32 spots (**Figure 3**). These spots were next analyzed by LC-MS-MS/MS after trypsin digestion, using the MASCOT search engine to analyze MS data and identify proteins from primary sequence databases. The identified immunopositive proteins are listed in **Table 1**. However, these proteins should be considered putatively nitrated until the nitration sites have been identified by sequence analysis.

**Table 1:** Nitrated proteins in potato leaves inoculated with *Phytophthora infestans* isolated from PVDF membrane and identified by LC-MS-MS/MS. Asterisks indicate proteins that have previously been functionally confirmed as nitrated in animal or plant systems.

No.	Protein name	Score	Protein ID	MW/PI	Functional category
S1	Photosystem I iron-sulfur center	38	Q2VEC9	9,038/6,68	photosynthesis
S2	Actin-85C (Fragment) *	226	P30170	21, 695/5,89	cytoskeleton organization
S3	Actin-42 (Fragment) *	445	P93587	36,854/5,44	cytoskeleton organization
S4	Actin-58 *	576	P30167	41,786/5,46	cytoskeleton organization
S5	Threonine dehydratase biosynthetic, chloroplastic (Fragment)	66	P31212	39,088/4,71	amino acid metabolism
S6	ERBB-3 BINDING PROTEIN 1	35	M1CZC0	42,740/6,26	cell signaling and regulation
S7	S-adenosylmethionine synthase 1 S-adenosylmethionine synthase 2	409 260	Q307Y9 Q38JH8	43,216/5,52 42,702/5,67	amino acid metabolism amino acid metabolism
S8	Stearoyl-[acyl-carrier-protein] 9-desaturase, chloroplastic	50	P46253	44,538/6,37	lipid metabolism
S9	Formate dehydrogenase, mitochondrial	533	Q07511	42,038/6,64	energy metabolism
S10	Acetyl-coenzyme A carboxylase carboxyl transferase subunit beta, chloroplastic *	54	Q2VEG8	55,596/5,07	lipid metabolism
S11	Cytochrome c1-1, heme protein, mitochondrial * Cytochrome c1-2, heme protein, mitochondrial (Fragment) *	27 27	P25076 P29610	35,159/6,72 28,592/5,29	energy metabolism energy metabolism
S12	Actin-97 * Actin-100 *	1308 1192	P30171 P30173	41,643/5,31 41,644/5,23	cytoskeleton organization cytoskeleton organization
S13	4-alpha-glucanotransferase, chloroplastic/amyloplastic	47	Q06801	64, 951/5,28	carbohydrate metabolism
S14	Bifunctional L-3-cyanoalanine synthase/cysteine synthase 1, mitochondrial	457	Q76MX2	38,201/6,86	amino acid metabolism
S15	Isovaleryl-CoA dehydrogenase, mitochondrial	67	Q9FS87	45,264/7,96	lipid metabolism
S16	Pyruvate dehydrogenase E1 component subunit alpha, mitochondrial Isocitrate dehydrogenase [NADP]	1730 80	P52903 P50217	43,228/7,63 46,792/6,54	energy metabolism energy metabolism
S17	Glucose-1-phosphate adenylyltransferase small subunit, chloroplastic/amyloplastic	28	P23509	57,240/6,73	carbohydrate metabolism
S18	Oxygen-evolving enhancer protein 1, chloroplastic (OEE1)	987	P26320	35,389/5,84	photosynthesis
S19	Light-induced protein, chloroplastic Photosystem II protein D1	320 122	P80471 Q2VEJ6	35,635/5,26 38,951/5,12	photosynthesis photosynthesis
S20	Glucan endo-1,3-beta-glucosidase, basic isoform 2 Glucan endo-1,3-beta-glucosidase, basic isoform 1 (Fragment) Glucan endo-1,3-beta-glucosidase, basic isoform 3 (Fragment)	820 738 669	P52401 P52400 P52402	39,759/6,32 37,022/6,67 36,182/6,67	stress response stress response stress response
S21	Ribulose bisphosphate carboxylase large chain	564	P25079	52,944/6,55	photosynthesis
S22	ATP synthase subunit beta, chloroplastic	5440	Q2VEH0	53,508/5,35	energy metabolism
S23	2-methylacyl-CoA dehydrogenase, mitochondrial	76	Q9FS88	45,086/7,49	lipid metabolism
S24	Heat shock 70 kDa protein, mitochondrial *	64	M1AQ30	65,105/5,32	stress response
S25	Leucine aminopeptidase, chloroplastic	627	P31427	60,122/5,94	proteases and protease inhibitors
S26	Heat shock 70 kDa protein, mitochondrial *	114	Q08276	73,077/6,37	stress response
S27	Catalase isozyme 2 * Catalase isozyme 1 *	277 267	P55312 P49284	56,451/6,56 56,370/6,56	stress response stress response
S28	Probable glutathione S-transferase *	85	P32111	25,056/5,31	stress response
S29	Fructose-1,6-bisphosphatase, cytosolic	535	P46276	37,311/5,80	carbohydrate metabolism
S30	NAD(P)H-quinone oxidoreductase subunit 2 A, chloroplastic	117	P0CD48	56,627/5,20	energy metabolism
S31	Linoleate 13S-lipoxygenase 2-1, chloroplastic (Lipoxygenase 2-1) *	158	O24370	101,937/6,10	stress response
S32	N-carbamoylputrescine amidase	160	Q3HVN1	33,405/5,87	amino acid metabolism
S33	Cysteine synthase chloroplastic/chromoplastic	256	P81154	34,342/6,27	amino acid metabolism
S34	Calmodulin-5/6/7/8 * Calmodulin-2/4 (Fragment) * Calmodulin-1 *	49 49 49	Q7DMN9 Q7DMP0 P13868	16,848/4,11 13,986/4,21 16,904/4,15	cell signaling and regulation cell signaling and regulation cell signaling and regulation
S35	Actin-75*	576	P30169	41,928/5,29	cytoskeleton organization
S36	Fructokinase Probable UDP-arabinopyranose mutase 2	329 75	P37829 Q8RU27	33,765/5,47 41,603/5,71	carbohydrate metabolism carbohydrate metabolism
S37	NAD(P)H:quinone oxidoreductase Aspartic protease inhibitor 8 Aspartic protease inhibitor 9 Serine protease inhibitor 1	188 80 80 80	Q8H9D2 P17979 P58521 P58514	24,486/6,17 24,588/6,26 20,631/6,51 24,362/6,70	stress response proteases and protease inhibitors proteases and protease inhibitors proteases and protease inhibitors
S38	14-3-3-like protein * 14-3-3-like protein 16R *	123 123	Q41418 P93784	29,395/4,71 28,936/4,72	cell signaling and regulation cell signaling and regulation
S39	ATP synthase subunit alpha, chloroplastic Mitochondrial-processing peptidase subunit alpha	641 270	Q27S65 P29677	55,411/5,14 54,677/5,71	energy metabolism transport and membrane proteins
S40	Ribulose bisphosphate carboxylase small chain C, chloroplastic	97	P10647	20,368/6,73	photosynthesis
S41	Probable sucrose-phosphate synthase	24	Q43845	118,292/6,07	carbohydrate metabolism
S42	Transketolase, chloroplastic	115	Q43848	79,992/5,94	carbohydrate metabolism
S43	Alpha-glucan water dikinase, chloroplastic (Starch-related R1 protein)	28	Q9AWA5	163,237/5,85	carbohydrate metabolism
S44	14-3-3-like protein RA215 *	52	Q43643	29,395/4,71	cell signaling and regulation
S45	Glucose-1-phosphate adenylyltransferase large subunit 1 (Fragment)	199	Q00081	52,253/6,09	carbohydrate metabolism

**Figure 4:**
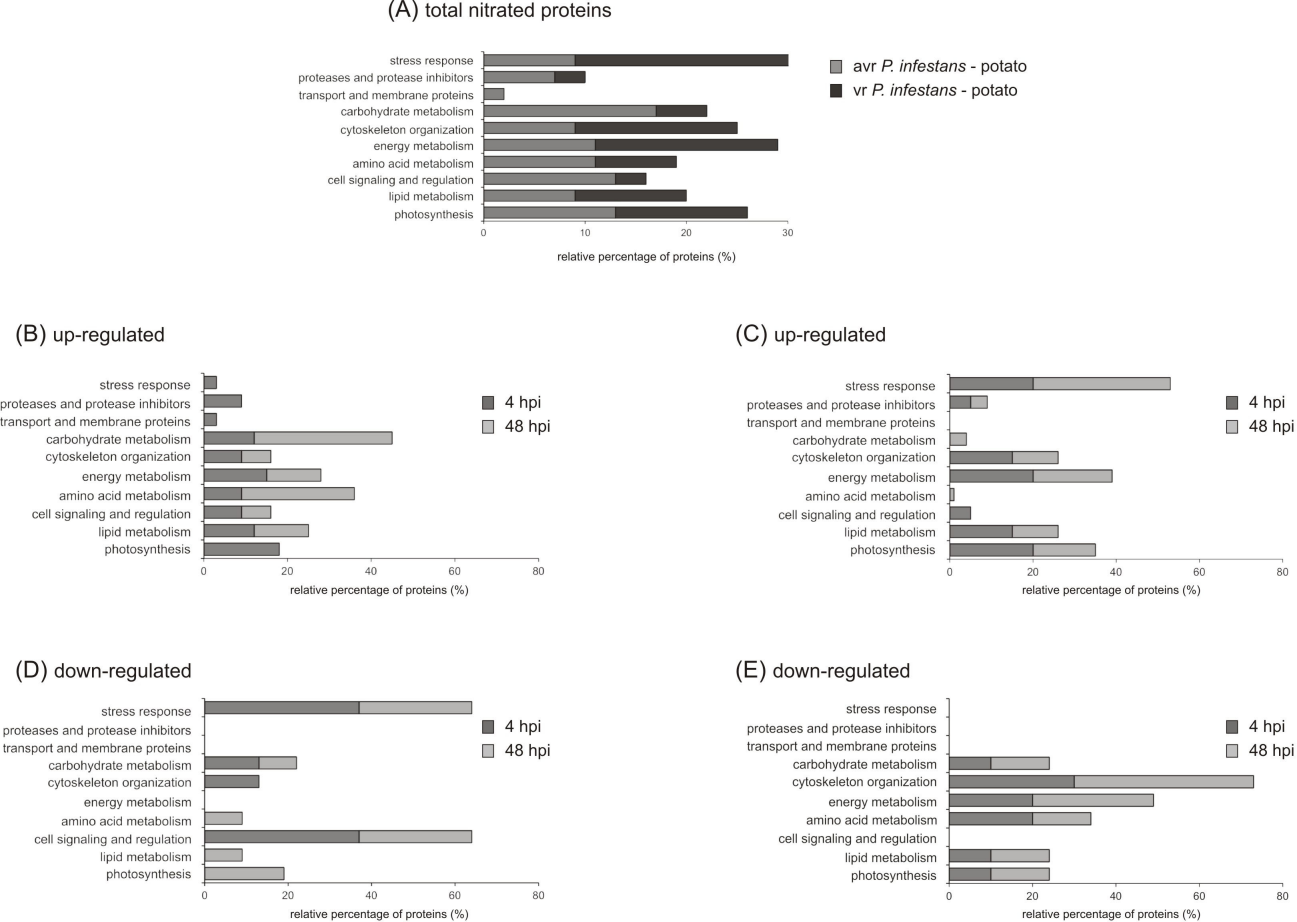
Functional classification and distribution of 45 nitrated protein spots identified by LC–MS–MS/MS analysis in leaves of potato genotypes at 4 and 48 h challenge-inoculated with *Phytophthora infestans*. **(A)** A total of differentially expressed proteins was identified in both potato-*P. infestans* interactions. Up- **(B)**, and down- **(D)** regulated proteins detected during avr *P. infestans* interaction. Up- **(C)**, and down- **(E)** regulated proteins detected during vr *P. infestans*. The area for each group shows the relative percentage (%) of proteins in that group.

Additionally, the identified proteins were classified into 10 functional categories corresponding to different biological processes (according to the Uniprot database), as shown in **Figure 4**. The categories containing the highest number of identified protein candidates for nitration in both potato genotypes after *P. infestans* challenge are related to cytoskeleton organization (10-16%), energy metabolism (11-18%), amino acid metabolism (11%), and photosynthesis (13%) (**Figure 4A**). Importantly, in potato-avr *P. infestans* interaction, the proteins associated with carbohydrate metabolism (18%) constituted the most-represented functional category in addition to proteins involved in cell signaling and regulation (15%) (**Figures 4A, B**). In turn, in the potato-vr *P. infestans* interaction, a majority of the identified potentially nitrated proteins were involved in stress response (24%) (**Figures 4A, C**).

**Figure 5:**
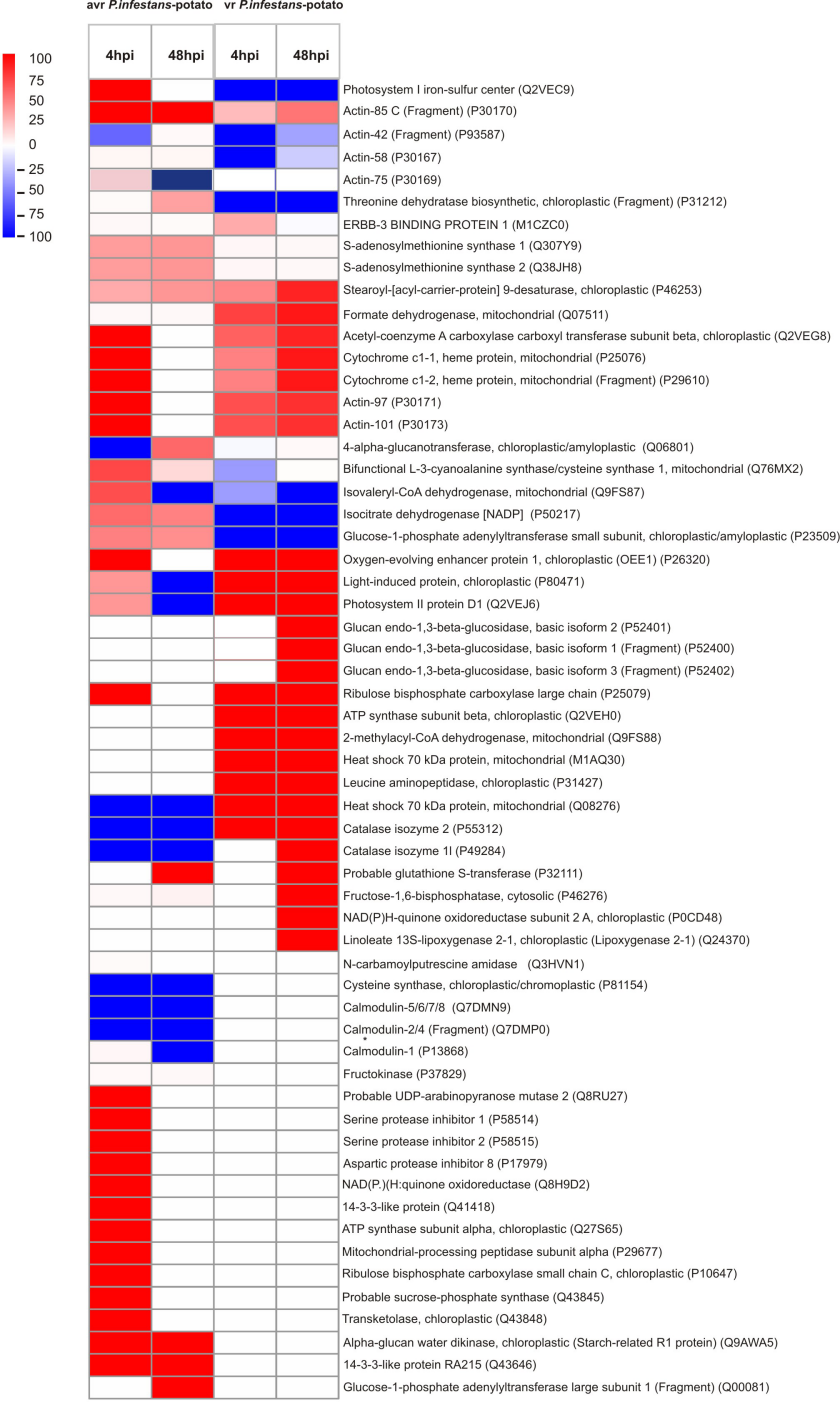
Heatmap of protein tyrosine nitration patterns in leaves of potato genotypes at 4 and 48 h post-inoculation with *Phytophthora infestans*. Unique and shared proteins with significant changes in nitration levels between genotypes and time points were used. Data represent significant shifts in tyrosine nitration intensity compared to mock-inoculated controls.

As shown in **Figure 5**, the comparative analysis of differentially expressed protein spots revealed distinct nitration patterns, depending on both the potato genotype and the time after pathogen inoculation. Potato-avr *P. infestans* interaction resulted in the identification of 31 spots at 4 hpi and 19 spots at 48 hpi, respectively (**Figure 2**). The fifteen protein spots were found to be common for both time after pathogen inoculation (s2-s9, s14, s16-s17, s23, s30, s36, s43-s44); in turn, as many as 14 spots (s1, s10-s12, s15, s18-s19, s21, s37-s42) were specific to 4 hpi and 3 (s13, s29, s45) spots were specific to 48 h (**Figure 2**). Importantly, 5 out of 31 spots appearing at 4 hpi were exclusively nitrated in leaves of resistant potato cultivar (s37-s39, s40, s42) (**Figure 2** in red color). These 5 unique candidates for nitration, among others, included serine protease inhibitor 1 (P58514), serine protease inhibitor 2 (P58515), 14-3-3-like protein (Q41418), and 14-3-3-like protein 16R (P93784) (**Figure 5**, **Table 1**). Moreover, detailed analyses of the nitroproteome maps obtained from mock- and *P. infestans*-inoculated leaves revealed that three spots (s27, s28, s34) were no longer detected at 4 and 48 hpi (**Figure 2**). Interestingly, these absent nitrated proteins included those mainly involved in cell signaling and stress responses (**Figures 2**, **5**; **Table 1**). Abundance profiles of nitrated proteins at 4 hpi showed that 7 protein spots (s7–s18, s14-s17, s19) were upregulated and 16 appeared *de novo* (s1-s2, s10-s12, s18, s21, s23, s37-s44) (**Figure 2**); however, only 9 of them were common with those detected at 48 hpi (**Figure 2**). These highly abundant spots included proteins involved in carbohydrate, amino acid, and lipid metabolism (**Figure 4**, **Table 1**). Additionally, several photosynthesis-related proteins, including PSI subunit PsaC (Q2VEC9), Rubisco-associated proteins (P25079), ATP synthase subunits (Q2VEH0), and the oxygen-evolving enhancer protein (P26320), were nitrated at 4 hpi. In turn, antioxidant enzymes such as catalases (P55312, P49284) and glutathione S-transferases (P32111) were identified as downregulated at both 4 hpi and 48 hpi. Interestingly, after 48 hpi in the resistant genotype, the expression of most proteases, photosynthetic and signaling proteins, and proteins involved in cytoskeleton organization, carbohydrate metabolism, and energy production was diminished (**Figure 4**).

In contrast, the qualitative nitroproteome analysis of the potato-vr *P. infestans* interaction showed a different pattern of tyrosine-nitrated proteins, encompassing a broader spectrum of upregulated proteins observed at 48 hpi, in comparison to those observed at the same time point in potato-avr *P. infestans* interaction (**Figures 2**-**5**). Nitroproteome maps revealed 18 spots at 4 hpi and 24 at 48 hpi (**Figure 3**). Seventeen protein spots were commonly upregulated at both time points after pathogen challenge (s2, s6-s12, s18-s19, s21-s27), and these proteins were associated with photosynthesis, metabolism, signal transduction, and stress (**Figure 5**). As many as 6 (s20, s28-s32) spots were specific for 48 hpi, and 3 of them (s20, s31, and s32) were indicated as exclusively nitrated in potato-vr *P. infestans* at 48 hpi (**Figure 3** in red color). Importantly, among specific nitration candidates, we identified several proteins involved in stress responses, including three isoforms of glucan endo-1,3-beta-glucosidase (**Figure 5**, **Table 1**).

### β-1,3-glucanase expression is differentially modulated in potato genotypes challenged by the pathogen

**Figure 6:**
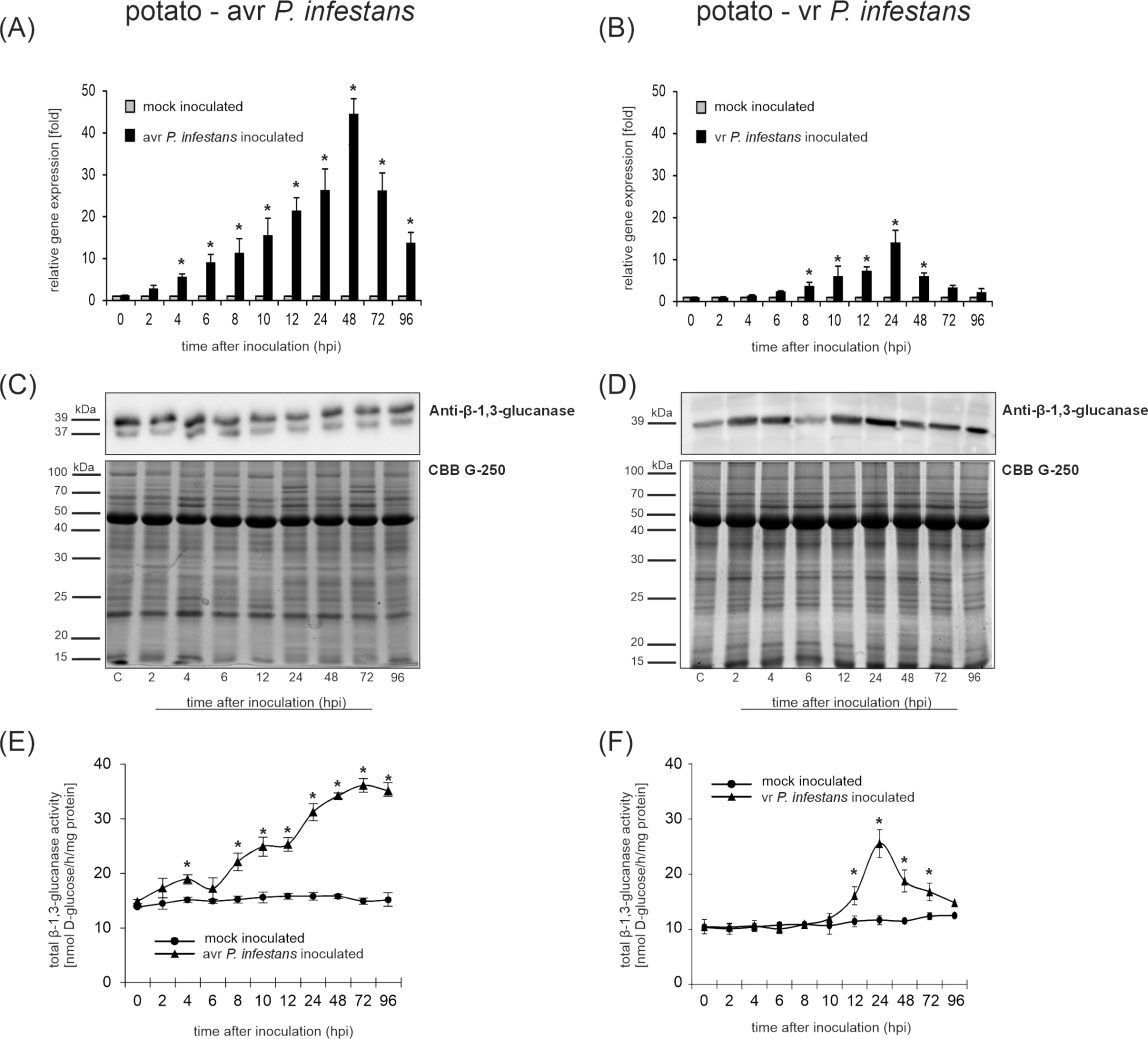
Analysis of β-1,3-glucanase gene expression, protein accumulation, and enzymatic activity in leaves of potato genotypes challenged with *Phytophthora infestans*. Transcript levels determined by qRT-PCR in resistant **(A)** and susceptible **(B)** potato inoculated with *P. infestans*. Protein accumulation in leaves of the resistant **(C)** and susceptible **(D)** genotypes following challenge inoculation with the pathogen. Equal amounts of protein (25 µg per lane) were loaded onto SDS–PAGE gels, and Coomassie Brilliant Blue (CBB) staining was used as a loading control. Enzymatic activity of β-1,3-glucanases was measured spectrophotometrically using laminarin as substrate in resistant **(E)** and susceptible **(F)** potato after inoculation. Values represent the mean ± SD of at least three independent experiments (n = 9). Asterisks indicate values that differ significantly from mock-inoculated (control) potato leaves at *P < 0.05.

Since β-1,3-glucanases (*PR-2*) catalyze the hydrolysis of glucans, which are a major component of the oomycete cell wall, their increased enzymatic activity may limit *P. infestans* tissue colonization. Therefore, the regulation of the potato β*-*1,3-glucanase expression was examined at the transcript, protein, and enzymatic activity levels in response to *P. infestans*. Based on the quantitative real-time PCR data, it was found that *PR-2* gene expression was rapidly and strongly activated only in potato-avr *P. infestans* interaction (**Figure 6A**). The expression of *PR-2* started to rise at 4 hpi and then gradually increased to reach the highest amount (over 40-fold increase in comparison to mock-inoculated leaves) at 48 hpi, and the mRNA level remained elevated till 96 hpi (**Figure 6A**). In contrast, in the potato-vr *P. infestans* interaction, robust *PR-2* transcript accumulation was observed only at the late phase (24 hpi). However, the *PR-2* mRNA was significantly upregulated since 12 hpi (**Figure 6B**).

Furthermore, the enhanced expression of the *PR-2* gene in the potato-avr *P. infestans* interaction was correlated in time with protein accumulation (**Figure 6C**) and an early increase in β-1,3-glucanase activity (**Figure 6E**). In turn, potato-vr *P. infestans* interaction displayed a less intense activation of PR-2 expression (**Figures 6D, F**).

### Peroxynitrite modulates PR-2 expression and enzymatic activity in potato leaves challenged with *P. infestans*

**Figure 7:**
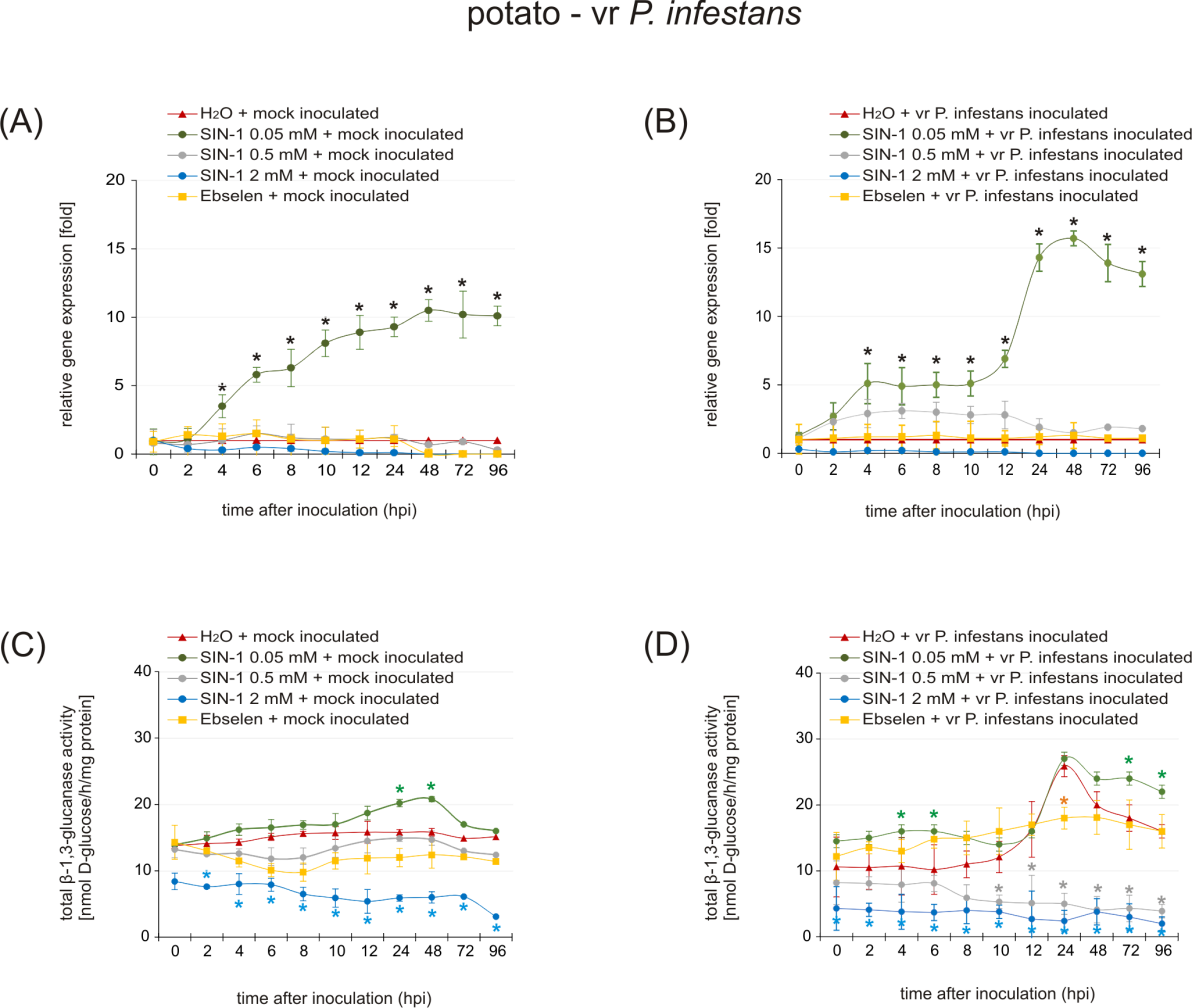
The effect of susceptible potato pretreatment with different concentrations (ranging from 0.05 mM to 2 mM) of peroxynitrite donor SIN-1, followed by challenge inoculation with *Phytophthora infestans* on PR-2 gene expression at the level of transcript **(A, B)**, and enzyme activity **(C, D)**. The analyses were performed at 0, 2, 4, 6, 12, 24, 48, 72, and 96 h after challenge inoculation. Asterisks indicate values that differ significantly from H_2_O mock-inoculated leaves **(A, C)** and H_2_O – vr *P. infetstans* inoculated leaves **(B, D)** at P < 0.05 (*).

Because most analyses of plants have shown that nitration usually causes a loss of protein function (Kolbert et al., 2017), the following experiments determine whether and to what extent endogenous nitration of β-1,3-glucanase regulates its activity. The analysis was performed using the susceptible potato genotype pretreated with different concentrations (0.05–2 mM) of the peroxynitrite donor SIN-1 (**Figures 7A–D**), which has been previously shown to mediate tyrosine nitration in potato plants (Arasimowicz-Jelonek et al., 2016; Izbiańska et al., 2018). Interestingly, a lower concentration (0.05 mM) of SIN-1 effectively induced PR-2 mRNA accumulation in healthy leaves starting at the 4th hour (**Figure 7A**). Moreover, the quantitative RT-PCR data revealed that leaves enriched with ONOO^–^ and subsequently challenged-inoculated showed an earlier and stronger induction of PR-2 than H_2_O-pretreated inoculated leaves (**Figure 7B**). Simultaneously, the activity of *PR-2* began to rise already at 4 hpi in leaves sequentially treated with 0.05 mM SIN-1 and *P. infestans* (**Figure 7C**). In turn, the higher ONOO^–^ concentrations (0.5 mM to 2 mM) markedly inhibited transcript accumulation and enzyme activity (**Figures 7A–D**). Importantly, potato leaves lacking ONOO^–^ due to ebselen pretreatment did not show significant expression of the *PR-2* gene after challenge inoculation (**Figures 7A–D**).

### Peroxynitrite inhibits recombinant potato β-1,3-glucanase activity in a dose-dependent manner

**Figure 8:**
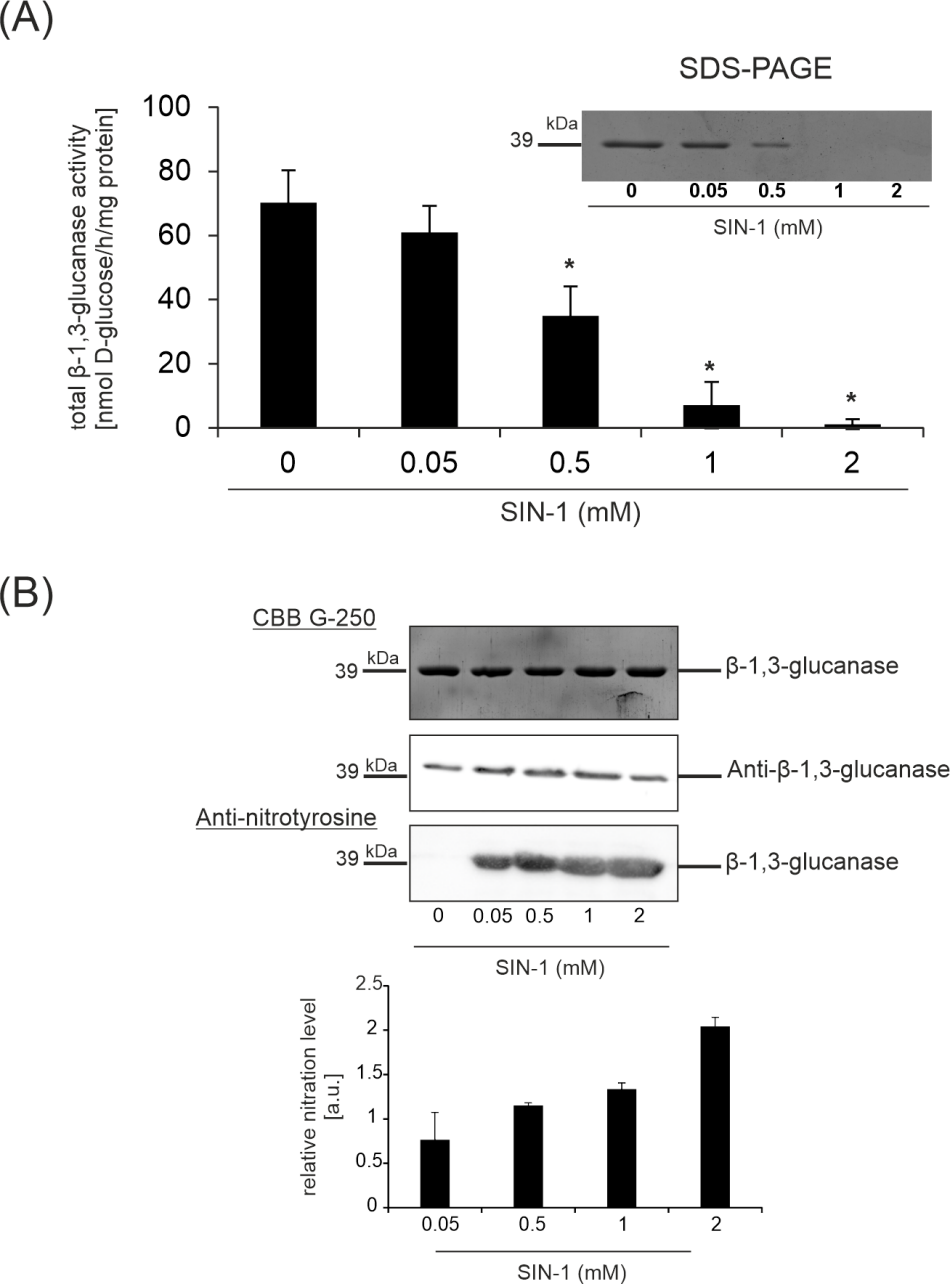
Effect of SIN-1 (ONOO^–^ donor) on recombinant β-1,3-glucanase activity **(A)**, protein accumulation and tyrosine nitration **(B)**. Samples were incubated at varying concentrations of SIN-1 (0.05–2 mM). Equal amounts of recombinant protein (1 µg per lane) were loaded onto the SDS-PAGE gel. Coomassie Brilliant Blue (CBB) staining served as a loading control.

To further evaluate whether β-1,3-glucanase nitration directly modulates enzymatic functions, the recombinant potato β-1,3-glucanase was treated with the ONOO^–^ donor SIN-1 at different concentrations (0.05–2 mM). As shown in **Figure 8A**, ONOO^–^ inhibits PR-2 activity in a dose-dependent manner, ranging from 30% at 0.05 mM SIN-1 to 98% at 2 mM SIN-1. To confirm that the observed inhibition was associated with nitration, an immunoblot analysis was performed on the *in vitro*-nitrated PR-2. As demonstrated in **Figure 8B**, the intensity of the nitrated PR-2 immunoreactivity with the antibody against nitrotyrosine increased, with nitration reaching its highest level with 2 mM SIN-1. Commercial nitrated bovine serum albumin (NO_2_-BSA) was used as a positive control (**Supplementary Figure 1**). Before the activity assay, the purity and equal loading of the recombinant PR-2 protein were verified by SDS-PAGE followed by Coomassie Brilliant Blue (CBB) staining (**Figure 8B**). A single, distinct band at the expected molecular weight was observed across all lanes, confirming protein stability and equal loading throughout the experiment.

### In silico identification of the active-site architecture in potato β-1,3-glucanase revealed the presence of highly conserved tyrosine residues

To gain deeper insight into the potential tyrosine nitration target, the tertiary structure of potato β-1,3-glucanase was modeled using the ortholog from *Hevea brasiliensis* as a template, which shares 64% identity (PDB ID: 4hpg.1.A). The quality assessment of the obtained model showed that 87% of the residues had a QMEANDisCo score above 0.2, and 97.1% were located in the most favored regions of the Ramachandran plot, with only 0.32% in the disallowed regions. The overall MolProbity score was 0.96, with a clashscore of 1.03, and only three residues in unfavorable non-local atomic interaction energy were located in loop regions. The global QMEAN score of -0.92 and the QMEANDisCo global value of 0.87 ± 0.05 confirmed the good stereochemical quality of the tertiary-structure model, comparable to experimentally determined proteins of similar size. The evolutionary conservation of tyrosine residues was assessed using the ConSurf server (Ashkenazy et al., 2016) based on 100 homologous sequences identified by BLASTP. Each tyrosine was further characterized in terms of amino acid content within a nine-residue window of linear sequence around the tyrosine (primary structure), solvent accessible surface area (ASA), secondary structure organization, and residues within 5 Å from the atom susceptible to undergoing nitration. The 15 identified tyrosine residues were distributed across distinct secondary structural elements: Tyr31, Tyr59, Tyr169, Tyr203, and Tyr335 were located in flexible coil or turn regions; Tyr114, Tyr236, and Tyr306 were found in β-strands; and Tyr48, Tyr142, Tyr205, Tyr208, Tyr219, Tyr248, and Tyr284 resided within α-helices (**Supplementary Table 3**).

**Figure 9:**
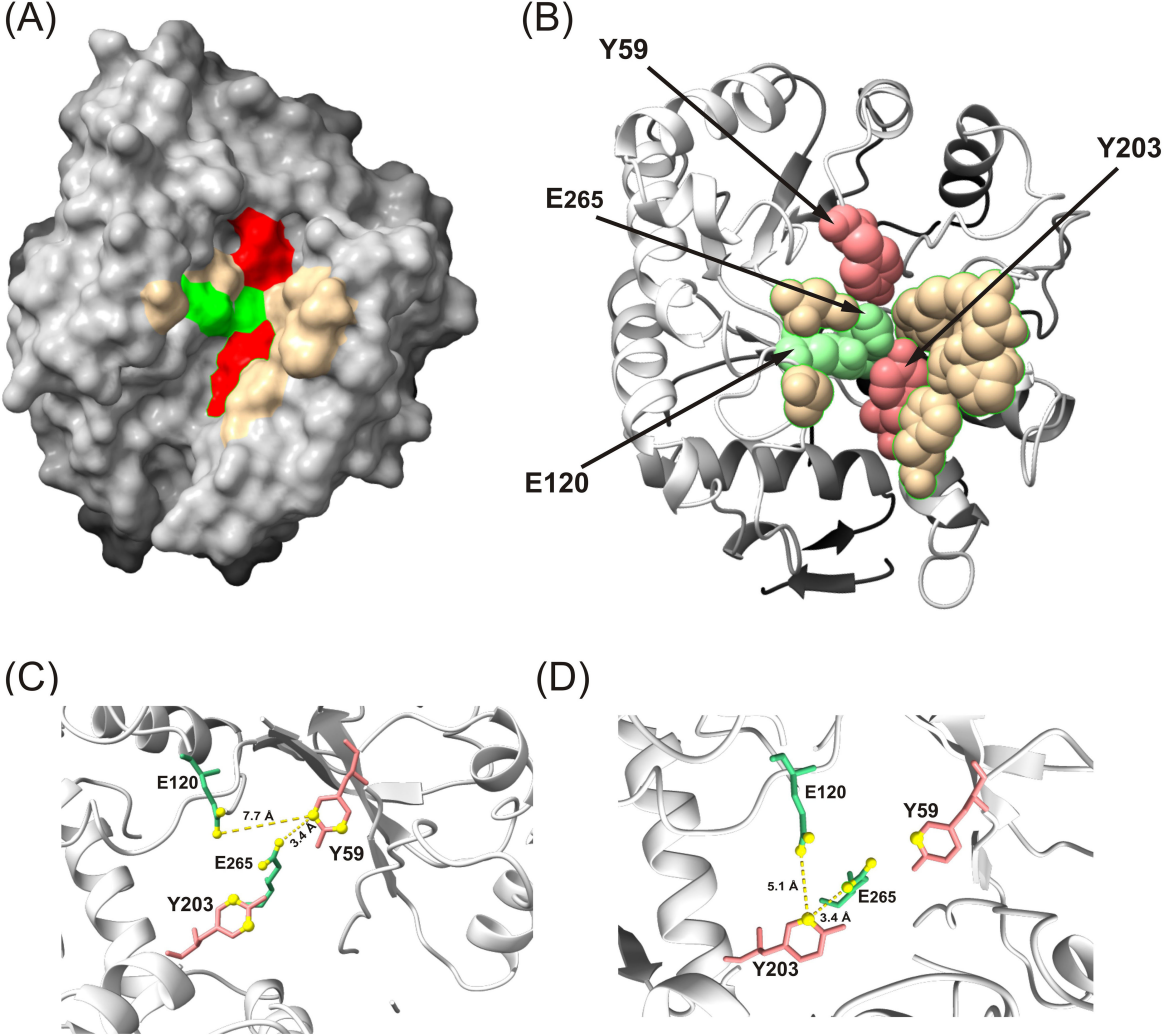
Structure of potato β-1,3-glucanase. The tertiary structure was generated by homology modeling, based on the coordinates of β-1,3-glucanase from *Hevea brasiliensis* (PDB ID: 4HPG, chain A). Surface active pocket identification by CASTp server: **(A)** Light Green, Red, and light yellow color boxes highlight the amino acid residues present in the binding site. **(B)** Shows the Tyr 59 and Tyr 203 in the catalytic pocket of β-1,3-glucanase. Calculated distances between the two Tyr residues (in Å) and the catalytic acid (E120) or the nucleophile (E265) **(C, D)**. Yellow dashed lines indicate distances between the residues.

Protein topography analysis using the CASTp 3.0 server (Ye et al., 2024) revealed two major surface pockets in the β-1,3-glucanase structure. The second pocket corresponded to the predicted catalytic cavity, containing the highly conserved Glu120 (acid/base) and Glu265 (nucleophile) residues, as annotated in UniProt (P52401). Importantly, two conserved tyrosine residues, Tyr59 and Tyr203, were located directly within the catalytic pocket (**Figures 9A, B**). Molecular distance calculations indicated that these residues are approximately 3.4 Å from the catalytic center (**Figures 9C, D**), suggesting they are potential targets for nitration-induced modulation of enzyme activity.

## Discussion

Progress in ‘omics’ and modeling tools has enabled the identification of protein tyrosine nitration as a crucial signaling mechanism in eukaryotes (Mata-Pérez et al., 2023). Although it is considered an irreversible process, some reversal of nitration involving denitrase activity has been characterized in animal cells (Deeb et al., 2013). However, a specific denitrase protein has not been identified, and no information is available in plants (Kolbert et al., 2017). Thus, elucidating the denitration process *in vivo* is still a challenge for scientists.

In higher plants, proteomic analyses of nitration have shown that a certain number of proteins are targets of this NO/ONOO^–^-mediated post-translational modification (PTM) during both physiological and stress conditions (e.g., Cecconi et al., 2009; Chaki et al., 2011; Lozano-Juste et al., 2011; Tanou et al., 2012; Arasimowicz-Jelonek et al., 2016; Gzyl et al., 2016). These experimentally proven results support the hypothesis that, *via* selective Tyr nitration, ONOO^–^ can be involved in a broad spectrum of signaling and regulatory processes (Arasimowicz-Jelonek and Floryszak-Wieczorek, 2011; Vandelle and Delledonne, 2011). Interestingly, accumulating evidence suggests that ONOO^–^ also plays a pivotal role in creating a cellular redox environment that promotes defense reactions (Durner et al., 1998; Alamillo and García-Olmedo, 2001). Recently, Arasimowicz-Jelonek et al. (2016); Izbiańska et al. (2018) showed that in potato, early and transient ONOO^–^ formation, correlated in time with elevated nitration of both proteins and RNA/mRNA, can lead to the activation of defense responses in resistant genotypes. Notably, significantly reduced numbers of cells undergoing programmed cell death (PCD) were observed in potato leaves pretreated with ONOO^–^ scavengers, confirming that ONOO^–^, along with NO and ROS, functions as a signaling mediator that facilitates the establishment of the hypersensitive response (HR).

In this work, an immunoassay for 3-nitrotyrosine detection was used to verify that only the potato-avr *P. infestans* interaction is accompanied by an early and transient tyrosine nitration, observed within the first 6 hpi (**Figure 1A**). In contrast, the vr *P. infestans* pathosystem exhibited a delayed and prolonged accumulation of nitrated protein with maximum levels coinciding with tissue colonization (**Figure 1B**). The presented data indicate genotype-specific dynamics of tyrosine nitration, which correlated in time with ONOO^–^ accumulation (Izbiańska et al., 2018). This temporal difference suggests distinct physiological roles for ONOO^–^ in both interactions. The early and transient nitration observed in the resistant genotype may act as a tightly regulated signaling event, functioning as a ‘molecular switch’ that activates defense pathways and restricts hypersensitive cell death. In contrast, the delayed and prolonged accumulation of nitrated proteins in the susceptible genotype may reflect a disruption of redox homeostasis. Under these conditions, late-stage nitration could indicate the development of nitrative stress, in which excessive ONOO^–^ accumulation may lead to non-specific modification or inactivation of metabolic and defense-related proteins, thereby facilitating pathogen colonization. Notably, Cecconi et al. (2009) found that in *Arabidopsis thaliana*, challenge inoculation with an avirulent bacterial pathogen, *Pseudomonas syringae* (pv. tomato), the disease resistance response is correlated with a modulation of nitrated protein involved in important cellular processes. It is worth noting that significant changes in the total pool of nitrated proteins were observed already at 4 and 8 hpi, supporting the hypothesis that Tyr-nitration may be a relevant physiological process in resistance (Cecconi et al., 2009). Nevertheless, the comprehensive understanding of which proteins are nitrated and how these modifications affect plant responses to pathogen attack and resistance mechanisms remains unclear (León, 2022).

In host cells, dynamic PTMs have emerged as powerful regulatory mechanisms that play an essential role in activating defense response and determining the outcome of the plant-pathogen interaction (Withers and Dong, 2017). However, identification of 3-nitrotyrosine-containing proteins and mapping of nitrated residues is a challenging task due to the limited incidence of this modification in biological samples (Batthyány et al., 2017; Bhat et al., 2024), in contrast with the large number of proteins modified by other post-translational events such as phosphorylation or glycosylation (Batthyány et al., 2017). Importantly, protein nitration, triggered by ONOO^–^, can alter protein structure, leading to reversible or irreversible changes in protein activity, and potentially playing a role in redox signaling, similar to phosphorylation (Sengupta and Bhattacharjee, 2016).

Technological advances in nitroproteome analysis have enabled the identification of more than 100 potentially nitrated proteins in plants under various physiological and stress conditions. Unfortunately, to date, only five published reports have described Tyr nitration under biotic stress conditions (Saito et al., 2006; Cecconi et al., 2009; Arasimowicz-Jelonek et al., 2016; Izbiańska et al., 2018). What is important is that only two of them are dedicated to identifying potential protein targets through mass spectrometry analysis. Twelve proteins undergoing Tyr nitration were identified in the leaves of *Arabidopsis* challenged with an avirulent bacterial pathogen, and they are involved in photosynthesis, nitrogen assimilation, ATP synthesis, the Calvin cycle, and glycolysis. Additionally, the preliminary studies conducted by Arasimowicz-Jelonek et al. (2016) using one-dimensional Western blot analysis showed that in potato leaves challenged with *Phytophthora infestans*, 40 nitrated proteins were detected at 24 and 48 hpi, including redox-related, signaling/regulatory, primary metabolic, and stress-related proteins. This study, using two-dimensional gel electrophoresis, identified over 45 potential protein Tyr-nitration candidates in the leaves of both potato genotypes at 4 and 48 hpi. The analysis of pathogen-induced protein expression revealed significant quantitative and functional differences in the nitrated protein profile, which depended on both the potato genotype and the time after pathogen inoculation (**Figures 2**-**5**). In the potato-avr *P. infestans* interaction, an early and transient nitration of proteins involved in cellular signaling, carbohydrate metabolism, and photosynthesis was observed at 48 hpi (**Figures 2**-**5**). Repression of photosynthesis is a common response to host-pathogen interactions (Berger et al., 2007; Jiang et al., 2023). During defense responses, plants often maintain energy balance by upregulating defense-related pathways while downregulating other metabolic processes, such as photosynthesis. This reduction in photosynthetic activity likely helps offset the increased energy expenditure required to activate defense mechanisms (Rojas et al., 2014). Furthermore, the observed nitration of enzymes in glycolysis and the tricarboxylic acid (TCA) cycle (**Figure 5**) may act as a rapid, post-translational regulatory mechanism to reprogram metabolic flux. This process may redirect carbon resources toward the pentose phosphate pathway, supporting NADPH production required for the oxidative burst and specialized metabolite biosynthesis (Zheng et al., 2025). Similarly, nitration of signaling proteins may act as a feedback mechanism that fine-tunes the intensity of the defense response and prevents excessive cellular damage (Kolbert et al., 2017).

In contrast, in the susceptible genotype, the pathogen induced an enhanced upregulation of nitrotyrosine-containing proteins, which are primarily involved in carbohydrate metabolism and defense responses (**Figures 3**-**5**). The enhanced nitration of these protein classes during compatible interactions may indicate the inhibition of important cellular pathways and reflect a cellular imbalance that leads to disease development. These results confirmed the earlier observations that, during the potato-vr *P. infestans* interaction, the pathogen can exploit tyrosine nitration as a strategy to disrupt host cell metabolism and neutralize defense proteins (Izbiańska et al., 2019). Interestingly, among the defense-related proteins that appeared at 48 hpi in the susceptible potato genotype (s20) was β-1,3-glucanase, a pathogenesis-related protein (PR).

It is well known that the accumulation of PR proteins in plant tissues provides an effective defense mechanism activated in response to various pathogens. Pathogenesis-related proteins were originally identified in tobacco after infection with tobacco mosaic virus (TMV), but have since been found in a wide variety of plant species, including potatoes (Wang et al., 2005). Among them, chitinases and β-1,3-glucanases are two important hydrolytic enzymes that are abundant in many plant species following infection by various pathogens (dos Santos and Franco, 2023). Their antifungal properties are primarily associated with the enzymatic degradation of cell wall components, such as chitin in many fungal pathogens and β-1,3-glucans in fungal-like oomycetes (Perrot et al., 2022; dos Santos and Franco, 2023). Since β-1,3-glucanase is responsible for the hydrolysis of glucans, the increased activity of this enzyme can potentially limit the *P. infestans* infestation. Therefore, in this study, the rapid and enhanced expression of PR-2 at the transcript and protein levels was observed only during the potato-avr *P. infestans* interaction (**Figures 6A, C, E**). In turn, susceptible potato leaves displayed a less intense activation of PR-2 expression in response to *P. infestans* (**Figures 6B, D, F**).

Interestingly, the first experimental reports on NO metabolism in plants showed that exogenous ONOO^–^ application increased expression of the PR-1 gene in tobacco leaves (Durner et al., 1998). Most recently, using a pharmacological approach, Arasimowicz-Jelonek et al. (2016) demonstrated that treating the susceptible potato genotype with ONOO^–^ induced up-regulation of key markers of plant defense against pathogens, including PR-1, PR-2, and PR-3. Moreover, pretreatment of the susceptible genotype with ONOO^–^ and subsequent inoculation with *P. infestans* slowed pathogen colonization of host tissues by accelerating and intensifying upregulation of genes encoding PRs (Arasimowicz-Jelonek et al., 2016). In general, little is known about the specific impact of NO-related PTMs on the activity and structure of PR proteins involved in defense responses. However, nitrated chitinase (PR-3) has been identified in *Arabidopsis* (Lozano-Juste et al., 2011), *Citrus aurantium* (Tanou et al., 2012), potato (Arasimowicz-Jelonek et al., 2016), and tobacco (Takahashi et al., 2016); in turn, β-1,3-glucanase (PR-2) has been found to undergo nitration in leaves of potato (Arasimowicz-Jelonek et al., 2016). However, as mentioned before, no information is available on the functional consequences of tyrosine nitration in this important class of PR proteins. To gain deeper insight into the mechanism by which the β-1,3-glucanase is modulated *in vivo*, the susceptible genotype was pretreated with varying concentrations of the peroxynitrite donor. SIN-1 effectively stimulated β-1,3-glucanase expression and activity in healthy leaves only at a lower concentration (**Figures 7A, C**). A similar effect was observed after sequential treatment with the donors and subsequent inoculation with the pathogen (**Figures 7B, D**). In turn, higher ONOO^–^ concentrations (0.5 mM to 2 mM) in both cases markedly inhibited enzyme activity (**Figures 7A–D**). These results indicate that, while low concentrations of ONOO^–^ act as signals for defense gene induction, its overaccumulation in susceptible tissues may lead to the functional inactivation of PR-2 through tyrosine nitration. This selective post-translational modification likely impairs the enzyme’s ability to hydrolyze the *P. infestans* cell wall. As a result, the susceptible genotype, despite expressing PR-2, fails to halt infection because the protein is present but functionally compromised. Overall, these findings suggest that tyrosine nitration of PR-2 represents a critical vulnerability that potentially facilitates pathogen colonization in susceptible potato cultivars.

To further evaluate whether β-1,3-glucanase nitration is responsible for the observed *in vivo* modulation of protein activity (**Figures 7C, D**), the ONOO^–^ donor was applied to the potato recombinant enzyme at varying concentrations (**Figure 8A**). The *in vitro* activity assay of this enzyme from potato leaves, conducted in the presence of elevated ONOO^–^ concentrations, revealed a dose-dependent inhibitory effect of ONOO^–^ on PR-2 activity (**Figure 8A**). Similar loss of function has been observed *in vivo* and *in vitro* with respect to several plant metabolic enzymes such as ascorbate peroxidase (Begara-Morales et al., 2014), S-adenosyl homocysteine hydrolase (Chaki et al., 2009), ferredoxin-NADP reductase (Chaki et al., 2011), carbonic anhydrase (Chaki et al., 2013), O-acetylserine(thiol)lyase A1 (Alvarez et al., 2011), monodehydroascorbate reductase (MDAR) (Begara-Morales et al., 2015) and cytosolic NADP-dependent isocitrate dehydrogenase (Begara-Morales et al., 2013) (**Supplementary Table 2**). This inhibition likely results from the covalent modification of critical tyrosine residues located within or near the catalytic pocket. Such modifications can induce steric hindrance or local conformational changes that potentially interfere with substrate binding or catalytic efficiency (Ferrer-Sueta et al., 2018).

**Figure 10:**
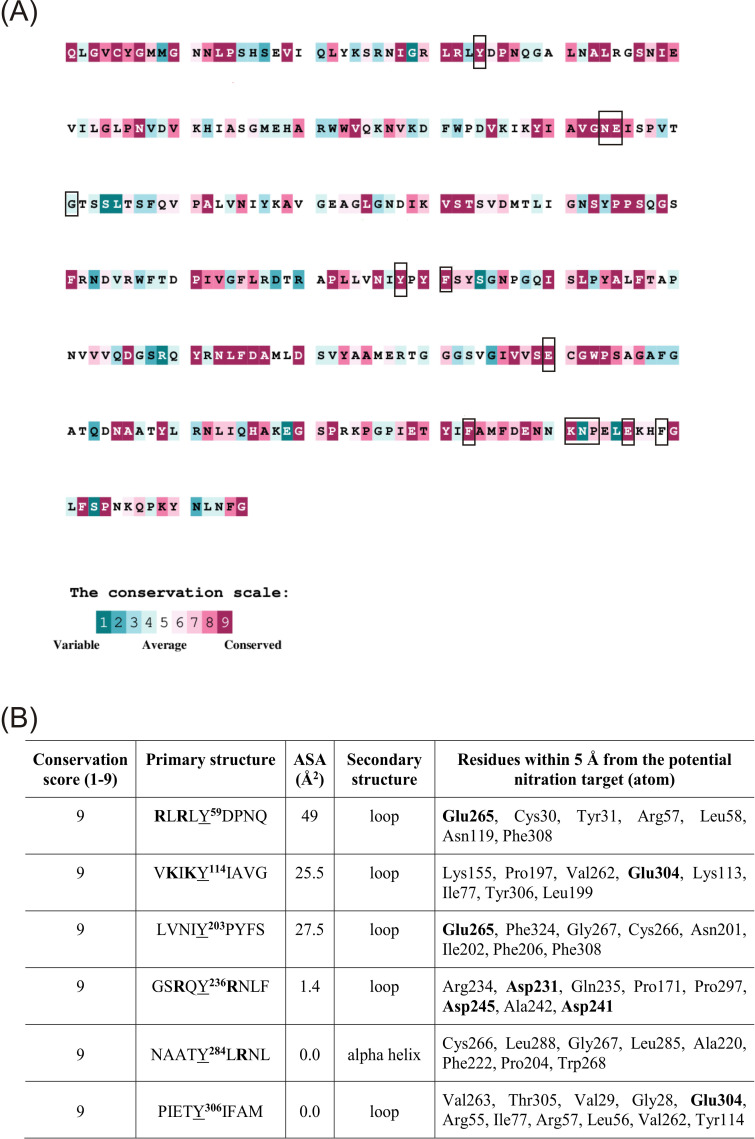
Evolutionary conservation of amino acid residues of potato β-1,3-glucanase (accession number P52401) was analyzed using the ConSurf server. Each residue was assigned a conservation grade ranging from 1 (variable) to 9 (highly conserved) based on a multiple-sequence alignment of homologous proteins. Residues located in the catalytic pocket are marked in black frames **(A)**. Characterization of the six highly conserved tyrosine (Y) residues (conservation score 9/9) present in potato β-1,3-glucanase in terms of amino acid content within a nine-residue window of linear sequence around the tyrosine (Primary structure), solvent-accessible surface area (ASA), secondary-structure organization, and residues within 5 Å from the two carbons susceptible to nitration. Residues meeting the nearby-residue criterion (basic amino acids in the immediate vicinity of the tyrosine residue within the primary sequence and/or the acidic residues within 5 Å from the atom susceptible to nitration) are shown in bold **(B)**.

For the identification of the Tyr residue/s potentially nitrated in the structure of potato β-1,3-glucanase, an *in silico* study was chosen. Since the nitration of tyrosine plays a significant role in protein modulation, it must be a selective process with a well-defined target that has been preserved throughout evolution. Thus, the evolutionary analysis of 100 sequences indicates that Tyr59, Tyr114, Tyr203, Tyr236, Tyr284, and Tyr306 are extremely well-conserved residues (**Figures 10A, B**), suggesting an important role. However, a systematic analysis of nitrated proteins in the mouse brain revealed that almost 60% of the nitrated tyrosine sites are predicted to be located at loop regions, where the solvent-accessible surface area is larger (Sacksteder et al., 2006). Tyrosines located in α-helices (H) generally participate in stabilizing the protein core and are often buried within the folded structure, thus being less reactive. Tyrosines situated within β-strands (E) are part of rigid sheet-like regions, potentially involved in inter-strand hydrogen bonding or hydrophobic packing, and may be partially accessible depending on orientation.

In contrast, tyrosines located in coil or loop regions (C) are more flexible, solvent-exposed, and are frequently involved in protein–protein interactions, substrate binding, or chemical modifications (Yeo et al., 2008). Therefore, the nitration of Tyr236, Tyr284, and Tyr306 seems unlikely because their solvent-accessible surface area is 1.4, 0.0, and 0.0 Å (ASA), respectively. Potential candidates with a large solvent-accessible surface area are Tyr59, Tyr114, and Tyr203. However, although all are good candidates, Tyr59 and Tyr203 were localized within the catalytic cavity in proximity (≤5 Å) to the catalytic residues Glu120 (acid/base) and Glu265 (nucleophile). Both were positioned in coil regions and exhibited high solvent accessibility, suggesting that they are flexible and exposed to the surrounding environment. However, Tyr203 fails the nearby-residue criterion, since neither of the basic amino acids is in its immediate vicinity within the primary sequence, although there is the acidic Glu265 residue within 3.4 Å from the atom susceptible to nitration (**Figure 9D**). Nevertheless, Tyr59 is proposed as the target for the nitration because it is located in a loop and fulfills both the solvent-accessible surface area and the nearby-residue criterion with Arg 55 and Arg 57 within a nine-residue window and Glu265 (nucleophile) within 3.4 Å from the atom susceptible to nitration (**Figure 9C**). Nitration of Tyr59 may sterically hinder substrate access to the active-site cleft or disrupt the proton-transfer dynamics mediated by Glu120 and Glu265, ultimately resulting in reduced enzymatic activity observed upon SIN-1 treatment.

## Conclusions

This study provides evidence that protein tyrosine nitration is a dynamic, genotype-dependent post-translational modification that contributes to the regulation of potato defense responses against *Phytophthora infestans*. Functional characterization of nitrated β-1,3-glucanase suggests that peroxynitrite-mediated nitration may selectively inhibit PR protein activity, with Tyr59 emerging as a potential regulatory site. Together, these findings support a dual role of protein nitration in plant immunity, linking transient modifications with defense activation and excessive nitration with pathogen-associated suppression of host resistance. While future studies, including mass spectrometry-based site identification and site-directed mutagenesis will be required to confirm the specific role of Tyr59, these results provide a solid biochemical and structural foundation for understanding the nitroproteome as an integral component of NO-dependent host–pathogen signaling.

## Data Availability

The raw data supporting the conclusions of this article will be made available by the authors, without undue reservation.

## References

[B1] Abeles F. B. Forrence L. E. (1970). Temporal and hormonal control of β-1,3-glucanase in Phaseolus vulgaris l. Plant Physiol. 45, 395–400. doi: 10.1104/pp.45.4.395 5427109 PMC396421

[B2] Alamillo J. M. García-Olmedo F. (2001). Effects of urate, a natural inhibitor of peroxynitrite-mediated toxicity, in the response of Arabidopsis thaliana to the bacterial pathogen pseudomonas syringae. Plant J. 25, 529–540. doi: 10.1046/j.1365-313x.2001.00984.x 11309143

[B3] Alvarez C. Lozano-Juste J. Romero L. C. García I. Gotor C. León J. (2011). Inhibition of arabidopsis o-acetylserine(thiol)lyase a1 by tyrosine nitration. J. Biol. Chem. 286, 578–586. doi: 10.1074/jbc.M110.147678 21047785 PMC3013017

[B4] Arasimowicz-Jelonek M. Floryszak-Wieczorek J. (2011). Understanding the fate of peroxynitrite in plant cells – from physiology to pathophysiology. Phytochemistry 72, 681–688. doi: 10.1016/j.phytochem.2011.02.025 21429536

[B5] Arasimowicz-Jelonek M. Floryszak-Wieczorek J. Izbiańska K. Gzyl J. Jelonek T. (2016). Implication of peroxynitrite in defence responses of potato to Phytophthora infestans. Plant Pathol. 65, 754–766. doi: 10.1111/ppa.12471 41875165

[B6] Arnold K. Bordoli L. Kopp J. Schwede T. (2006). The SWISS-MODEL workspace: a web-based environment for protein structure homology modelling. Bioinformatics 22, 195–201. doi: 10.1093/bioinformatics/bti770 16301204

[B7] Ashkenazy H. Abadi S. Martz E. Chay O. Mayrose I. Pupko T. . (2016). ConSurf 2016: an improved methodology to estimate and visualize evolutionary conservation in macromolecules. Nucleic Acids Res. 44, W344–W350. doi: 10.1093/nar/gkw408 27166375 PMC4987940

[B8] Bartesaghi S. Radi R. (2018). Fundamentals on the biochemistry of peroxynitrite and protein tyrosine nitration. Redox Biol. 14, 618–625. doi: 10.1016/j.redox.2017.09.009 29154193 PMC5694970

[B9] Batthyány C. Bartesaghi S. Mastrogiovanni M. Lima A. Demicheli V. Radi R. (2017). Tyrosine-nitrated proteins: proteomic and bioanalytical aspects. Antioxid. Redox Signal. 26, 313–328. doi: 10.1089/ars.2016.6787 27324931 PMC5326983

[B10] Begara-Morales J. C. Chaki M. Sánchez-Calvo B. Mata-Pérez C. Leterrier M. Palma J. M. . (2013). Protein tyrosine nitration in pea roots during development and senescence. J. Exp. Bot. 64, 1121–1134. doi: 10.1093/jxb/ert006 23362300 PMC3580824

[B11] Begara-Morales J. C. Sánchez-Calvo B. Chaki M. Mata-Pérez C. Valderrama R. Padilla M. N. . (2015). Differential molecular response of monodehydroascorbate reductase and glutathione reductase by nitration and S-nitrosylation. J. Exp. Bot. 66, 5983–5996. doi: 10.1093/jxb/erv306 26116026 PMC4566986

[B12] Begara-Morales J. C. Sánchez-Calvo B. Chaki M. Valderrama R. Mata-Pérez C. López-Jaramillo J. . (2014). Dual regulation of cytosolic ascorbate peroxidase (APX) by tyrosine nitration and S-nitrosylation. J. Exp. Bot. 65, 527–538. doi: 10.1093/jxb/ert396 24288182 PMC3904709

[B13] Begara-Morales J. C. Sánchez-Calvo B. Gómez-Rodríguez M. V. Chaki M. Valderrama R. Mata-Pérez C. . (2019). Short-term low temperature induces nitro-oxidative stress that deregulates the NADP-malic enzyme function by tyrosine nitration in Arabidopsis thaliana. Antioxid. (Basel). 8, 448. doi: 10.3390/antiox8100448 31581524 PMC6827146

[B14] Bellin D. Asai S. Delledonne M. Yoshioka H. (2013). Nitric oxide as a mediator for defense responses. MPMI 26, 271–277. doi: 10.1094/MPMI-09-12-0214-CR 23151172

[B15] Berger S. Sinha A. K. Roitsch T. (2007). Plant physiology meets phytopathology: plant primary metabolism and plant-pathogen interactions. J. Exp. Bot. 58, 4019–4026. doi: 10.1093/jxb/erm298 18182420

[B16] Bhat F. A. Mangalaparthi K. K. Ding H. Jain A. Hsu J.-S. Peterson J. A. . (2024). Exploration of nitrotyrosine-containing proteins and peptides by antibody-based enrichment strategies. Mol. Cell. Proteomics 23, 100733. doi: 10.1016/j.mcpro.2024.100733 38342410 PMC10950883

[B17] Bigeard J. Colcombet J. Hirt H. (2015). Signaling mechanisms in pattern-triggered immunity (PTI). Mol. Plant 8, 521–539. doi: 10.1016/j.molp.2014.12.022 25744358

[B18] Bleau J. R. Spoel S. H. (2021). Selective redox signaling shapes plant–pathogen interactions. Plant Physiol. 186, 53–65. doi: 10.1093/plphys/kiaa088 33793940 PMC8154045

[B19] Bradford M. M. (1976). A rapid and sensitive method for the quantitation of microgram quantities of protein utilizing the principle of protein-dye binding. Anal. Biochem. 72, 248–254. doi: 10.1016/0003-2697(76)90527-3 942051

[B20] Castillo M.-C. Lozano-Juste J. González-Guzmán M. Rodriguez L. Rodriguez P. L. León J. (2015). Inactivation of PYR/PYL/RCAR ABA receptors by tyrosine nitration may enable rapid inhibition of ABA signaling by nitric oxide in plants. Sci. Signal. 8, ra89. doi: 10.1126/scisignal.aaa7981 26329583

[B21] Cecconi D. Orzetti S. Vandelle E. Rinalducci S. Zolla L. Delledonne M. (2009). Protein nitration during defense response in Arabidopsis thaliana. Electrophoresis 30, 2460–2468. doi: 10.1002/elps.200800826 19598157

[B22] Chaki M. Valderrama R. Fernández-Ocaña A. M. Carreras A. López-Jaramillo J. Luque F. . (2009). Protein targets of tyrosine nitration in sunflower (Helianthus annuus L.) hypocotyls. J Exp Bot. 60(15), 4221–34. doi: 10.1093/jxb/erp263, PMID: 19717529

[B23] Chaki M. Valderrama R. Fernández-Ocaña A. M. Carreras A. Gómez-Rodríguez M. V. Pedrajas J. R. . (2011). Mechanical wounding induces a nitrosative stress by down-regulation of GSNO reductase and an increase in S-nitrosothiols in sunflower (Helianthus annuus) seedlings. J. Exp. Bot. 62, 1803–1813. doi: 10.1093/jxb/erq358 21172815 PMC3060671

[B24] Chaki M. Carreras A. López-Jaramillo J. Begara-Morales J. C. Sánchez-Calvo B. Valderrama R. . (2013). Tyrosine nitration provokes inhibition of sunflower carbonic anhydrase (β-CA) activity under high temperature stress. Nitric Oxide. 29, 30–3. doi: 10.1016/j.niox.2012.12.003, PMID: 23266784

[B25] Corpas F. J. Leterrier M. Begara-Morales J. C. Valderrama R. Chaki M. López-Jaramillo J. . (2013a). Inhibition of peroxisomal hydroxypyruvate reductase (HPR1) by tyrosine nitration. Biochim. Biophys. Acta 1830, 4981–4989. doi: 10.1016/j.bbagen.2013.07.002 23860243

[B26] Corpas F. J. Palma J. M. del Río L. A. Barroso J. B. (2013b). Protein tyrosine nitration in higher plants grown under natural and stress conditions. Front. Plant Sci. 4. doi: 10.3389/fpls.2013.00029 23444154 PMC3580390

[B27] Costa-Broseta Á. Castillo M. León J. (2020). Nitrite reductase 1 is a target of nitric oxide-mediated post-translational modifications and controls nitrogen flux and growth in arabidopsis. Int. J. Mol. Sci. 21, 7270. doi: 10.3390/ijms21197270 33019636 PMC7582248

[B28] Costa-Broseta Á. Castillo M. León J. (2021). Post-translational modifications of nitrate reductases autoregulates nitric oxide biosynthesis in arabidopsis. Int. J. Mol. Sci. 22, 549. doi: 10.3390/ijms22020549 33430433 PMC7827142

[B29] Deeb R. S. Nuriel T. Cheung C. Summers B. Lamon B. D. Gross S. S. . (2013). Characterization of a cellular denitrase activity that reverses nitration of cyclooxygenase. Am. J. Physiol. Heart Circ. Physiol. 305, H687–H698. doi: 10.1152/ajpheart.00876.2012 23792683 PMC3761327

[B30] dos Santos C. Franco O. L. (2023). Pathogenesis-related proteins (PRs) with enzyme activity activating plant defense responses. Plants (Basel). 12, 2226. doi: 10.3390/plants12112226 37299204 PMC10255391

[B31] Durner J. Wendehenne D. Klessig D. F. (1998). Defense gene induction in tobacco by nitric oxide, cyclic GMP, and cyclic ADP-ribose. Proc. Natl. Acad. Sci. U.S.A. 95, 10328–10333. doi: 10.1073/pnas.95.17.10328 9707647 PMC21508

[B32] Eisenberg D. Lüthy R. Bowie J. U. (1997). VERIFY3D: assessment of protein models with three-dimensional profiles. Methods Enzymol. 277, 396–404. doi: 10.1016/s0076-6879(97)77022-8 9379925

[B33] Ferrer-Sueta G. Campolo N. Trujillo M. Bartesaghi S. Carballal S. Romero N. . (2018). Biochemistry of peroxynitrite and protein tyrosine nitration. Chem. Rev. 118, 1338–1408. doi: 10.1021/acs.chemrev.7b00568 29400454

[B34] Floryszak-Wieczorek J. Arasimowicz-Jelonek M. (2016). Contrasting regulation of NO and ROS in potato defense-associated metabolism in response to pathogens of different lifestyles. PloS One 11, e0163546. doi: 10.1371/journal.pone.0163546 27695047 PMC5047594

[B35] Frishman D. Argos P. (1995). Knowledge-based protein secondary structure assignment. Proteins 23, 566–579. doi: 10.1002/prot.340230412 8749853

[B36] Galetskiy D. Lohscheider J. N. Kononikhin A. S. Popov I. A. Nikolaev E. N. Adamska I. (2011). Mass spectrometric characterization of photooxidative protein modifications in Arabidopsis thaliana thylakoid membranes. Rapid Commun. Mass. Spectrom. 25, 184–190. doi: 10.1002/rcm.4855 21154902

[B37] Gebhardt C. Ballvora A. Walkemeier B. Oberhagemann P. Schüler K. (2004). Assessing genetic potential in germplasm collections of crop plants by marker-trait association: a case study for potatoes with quantitative variation of resistance to late blight and maturity type. Mol. Breed. 13, 93–102. doi: 10.1023/B:MOLB.0000012878.89855.df 38124636

[B38] Gzyl J. Izbiańska K. Floryszak-Wieczorek J. Jelonek T. Arasimowicz-Jelonek M. (2016). Cadmium affects peroxynitrite generation and tyrosine nitration in seedling roots of soybean (Glycine max l.). Environ. Exp. Bot. 131, 155–163. doi: 10.1016/j.envexpbot.2016.07.009 41909469

[B39] Holzmeister C. Gaupels F. Geerlof A. Sarioglu H. Sattler M. Durner J. . (2015). Differential inhibition of arabidopsis superoxide dismutases by peroxynitrite-mediated tyrosine nitration. J. Exp. Bot. 66, 989–999. doi: 10.1093/jxb/eru458 25428993 PMC4321555

[B40] Hurkman W. J. Tanaka C. K. (1986). Solubilization of plant membrane proteins for analysis by two-dimensional gel electrophoresis. Plant Physiol. 81, 802–806. doi: 10.1104/pp.81.3.802 16664906 PMC1075430

[B41] Izbiańska K. Floryszak-Wieczorek J. Gajewska J. Gzyl J. Jelonek T. Arasimowicz-Jelonek M. (2019). Switchable nitroproteome states of Phytophthora infestans biology and pathobiology. Front. Microbiol. 10. doi: 10.3389/fmicb.2019.01516 31379758 PMC6647872

[B42] Izbiańska K. Floryszak-Wieczorek J. Gajewska J. Meller B. Kuźnicki D. Arasimowicz-Jelonek M. (2018). RNA and mRNA nitration as a novel metabolic link in potato immune response to Phytophthora infestans. Front. Plant Sci. 9. doi: 10.3389/fpls.2018.00672 29896206 PMC5987678

[B43] Jiang X. Walker B. J. He S. Y. Hu J. (2023). The role of photorespiration in plant immunity. Front. Plant Sci. 14. doi: 10.3389/fpls.2023.1125945 36818872 PMC9928950

[B44] Khan R. A. A. Najeeb S. Chen J. Wang R. Zhang J. Hou J. . (2023). Insights into the molecular mechanism of trichoderma stimulating plant growth and immunity against phytopathogens. Physiol. Plant 175, e14133. doi: 10.1111/ppl.14133 38148197

[B45] Kolbert Z. Feigl G. Bordé Á. Molnár Á. Erdei L. (2017). Protein tyrosine nitration in plants: present knowledge, computational prediction and future perspectives. Plant Physiol. Biochem. 113, 56–63. doi: 10.1016/j.plaphy.2017.01.028 28187345

[B46] Laskowski R. A. MacArthur M. W. Moss D. S. Thornton J. M. (1993). PROCHECK: a program to check the stereochemical quality of protein structures. J. Appl. Crystallogr. 26, 283–291. doi: 10.1107/S0021889892009944 41799321

[B47] León J. (2022). Protein tyrosine nitration in plant nitric oxide signaling. Front. Plant Sci. 13. doi: 10.3389/fpls.2022.859374 35360296 PMC8963475

[B48] Lozano-Juste J. Colom-Moreno R. León J. (2011). *In vivo* protein tyrosine nitration in Arabidopsis thaliana. J. Exp. Bot. 62, 3501–3517. doi: 10.1093/jxb/err042 21378116 PMC3130175

[B49] Madeira F. Madhusoodanan N. Lee J. Eusebi A. Niewielska A. Tivey A. R. N. . (2024). Using EMBL-EBI services via web interface and programmatically via web services. Curr. Protoc. 4, e1065. doi: 10.1002/cpz1.1065 38857087

[B50] Mata-Pérez C. Sánchez-Vicente I. Arteaga N. Gómez-Jiménez S. Fuentes-Terrón A. Oulebsir C. S. . (2023). Functions of nitric oxide-mediated post-translational modifications under abiotic stress. Front. Plant Sci. 14. doi: 10.3389/fpls.2023.1158184 37063215 PMC10101340

[B51] Melo F. Feytmans E. (1998). Assessing protein structures with a non-local atomic interaction energy. J. Mol. Biol. 277, 1141–1152. doi: 10.1006/jmbi.1998.1665 9571028

[B52] Méndez A. A. E. Mangialavori I. C. Cabrera A. V. Benavides M. P. Vázquez-Ramos J. M. Gallego S. M. (2020). Tyr-nitration in maize CDKA;1 results in lower affinity for ATP binding. Biochim. Biophys. Acta Proteins Proteom. 1868, 140479. doi: 10.1016/j.bbapap.2020.140479 32599297

[B53] Möller M. N. Denicola A. (2024). Diffusion of peroxynitrite, its precursors, and derived reactive species, and the effect of cell membranes. Redox Biochem. Chem. 9, 100033. doi: 10.1016/j.rbc.2024.100033 41909469

[B54] Orłowska E. Fiil A. Kirk H.-G. Llorente B. Cvitanich C. (2012). Differential gene induction in resistant and susceptible potato cultivars at early stages of infection by Phytophthora infestans. Plant Cell Rep. 31, 187–203. doi: 10.1007/s00299-011-1155-2 21965005

[B55] Pan S. Q. Ye X. S. Kuc J. (1991). A technique for detection of chitinase, beta-1,3-glucanase, and protein-patterns after a single separation using polyacrylamide-gel electrophoresis or isoelectrofocusing. Phytopathol. 81, 970–974. doi: 10.1094/Phyto-81-970 40211709

[B56] Perrot T. Pauly M. Ramírez V. (2022). Emerging roles of β-glucanases in plant development and adaptative responses. Plants (Basel). 11, 1119. doi: 10.3390/plants11091119 35567119 PMC9099982

[B57] Pfaffl M. W. (2001). A new mathematical model for relative quantification in real-time RT-PCR. Nucleic Acids Res. 29, e45. doi: 10.1093/nar/29.9.e45 11328886 PMC55695

[B58] Plich J. Tatarowska B. Lebecka R. Śliwka J. Zimnoch-Guzowska E. Flis B. (2015). R2-like gene contributes to resistance to Phytophthora infestans in Polish potato cultivar Bzura. Am. J. Potato. Res. 92, 350–358. doi: 10.1007/s12230-015-9437-9 41913934

[B59] Rojas C. M. Senthil-Kumar M. Tzin V. Mysore K. (2014). Regulation of primary plant metabolism during plant-pathogen interactions and its contribution to plant defense. Front. Plant Sci. 5. doi: 10.3389/fpls.2014.00017 24575102 PMC3919437

[B60] Sabadashka M. Nagalievska M. Sybirna N. (2021). Tyrosine nitration as a key event of signal transduction that regulates functional state of the cell. Cell Biol. Int. 45, 481–497. doi: 10.1002/cbin.11301 31908104

[B61] Sainz M. Calvo-Begueria L. Pérez-Rontomé C. Wienkoop S. Abián J. Staudinger C. . (2015). Leghemoglobin is nitrated in functional legume nodules in a tyrosine residue within the heme cavity by a nitrite/peroxide-dependent mechanism. Plant J. 81, 723–735. doi: 10.1111/tpj.12762 25603991 PMC4346251

[B62] Saito S. Yamamoto-Katou A. Yoshioka H. Doke N. Kawakita K. (2006). Peroxynitrite generation and tyrosine nitration in defense responses in tobacco BY-2 cells. Plant Cell Physiol. 47, 689–697. doi: 10.1093/pcp/pcj038 16556649

[B63] Sengupta S. Bhattacharjee A . (2016). Dynamics of Protein Tyrosine Nitration and Denitration: A Review. J Proteo Genomics 1(1), 105. doi: 10.15744/2576-7690.1.105

[B64] Sacksteder C. A. Qian W. J. Knyushko T. V. Wang H. Chin M. H. Lacan G. . (2006). Endogenously nitrated proteins in mouse brain: links to neurodegenerative disease. Biochemistry. 45(26), 8009–22. doi: 10.1021/bi060474w, PMID: 16800626

[B65] Takahashi M. Shigeto J. Izumi S. Yoshizato K. Morikawa H. (2016). Nitration is exclusive to defense-related PR-1, PR-3 and PR-5 proteins in tobacco leaves. Plant Signal. Behav. 11, e1197464. doi: 10.1080/15592324.2016.1197464 27301959 PMC4991344

[B66] Takahashi M. Shigeto J. Sakamoto A. Izumi S. Asada K. Morikawa H. (2015). Dual selective nitration in Arabidopsis: Almost exclusive nitration of PsbO and PsbP, and highly susceptible nitration of four non-PSII proteins, including peroxiredoxin II E. Electrophoresis 36, 2569–2578. doi: 10.1002/elps.201500145 26177577

[B67] Tanou G. Filippou P. Belghazi M. Job D. Diamantidis G. Fotopoulos V. . (2012). Oxidative and nitrosative-based signaling and associated post-translational modifications orchestrate the acclimation of citrus plants to salinity stress. Plant J. 72, 585–599. doi: 10.1111/j.1365-313X.2012.05100.x 22780834

[B68] Tian W. Chen C. Lei X. Zhao J. Liang J. (2018). CASTp 3.0: computed atlas of surface topography of proteins. Nucleic Acids Res. 46, W363–W367. doi: 10.1093/nar/gky473 29860391 PMC6031066

[B69] Trapet P. Kulik A. Lamotte O. Jeandroz S. Bourque S. Nicolas-Francès V. . (2015). NO signaling in plant immunity: a tale of messengers. Phytochemistry 112, 72–79. doi: 10.1016/j.phytochem.2014.03.015 24713571

[B70] Vandelle E. Delledonne M. (2011). Peroxynitrite formation and function in plants. Plant Sci. 181, 534–539. doi: 10.1016/j.plantsci.2011.05.002 21893249

[B71] Wang D. Weaver N. D. Kesarwani M. Dong X . (2005). Induction of protein secretory pathway is required for systemic acquired resistance. Science 308(5724), 1036–40. doi: 10.1126/science.1108791, PMID: 15890886

[B72] Withers J. Dong X. (2017). Post-translational regulation of plant immunity. Curr. Opin. Plant Biol. 38, 124–132. doi: 10.1016/j.pbi.2017.05.004 28538164 PMC5644497

[B73] Ye B. Tian W. Wang B. Liang J . (2024). CASTpFold: Computed Atlas of Surface Topography of the universe of protein Folds. Nucleic Acids Res. 52(W1), W194–W199. doi: 10.1093/nar/gkae415, PMID: 38783102 PMC11223844

[B74] Yeo W. S. Lee S. J. Lee J. R. Kim K. P . (2008). Nitrosative protein tyrosine modifications: biochemistry and functional significance. BMB Rep. 41(3), 194–203. doi: 10.5483/bmbrep.2008.41.3.194, PMID: 18377722

[B75] Zhao S. Fernald R. D. (2005). Comprehensive algorithm for quantitative real-time polymerase chain reaction. J. Comput. Biol. 12, 1047–1064. doi: 10.1089/cmb.2005.12.1047 16241897 PMC2716216

[B76] Zheng K. Martinez M. P. Bouzid M. Balparda M. Schwarzländer M. Maurino V. G. (2025). Regulation of plant glycolysis and the tricarboxylic acid cycle by posttranslational modifications. Plant J. 122, e70142. doi: 10.1111/tpj.70142 40185637 PMC11971034

