# Supplementary material for: Peroxynitrite-mediated tyrosine nitration modulates β-1,3-glucanase activity and potato defense against Phytophthora infestans
Source: Front Plant Sci. 2026 Apr 17;17:1796014. doi: 10.3389/fpls.2026.1796014 (PMC13133032; doi:10.3389/fpls.2026.1796014)

**Table S1.** Selected nitrated proteins identified in higher plants, for which the specific tyrosine nitration sites have been determined and the physiological effects of this post-translational modification (PTM) have been characterized.

| **Protein** | | **Effect** | | **Identified nitrated**  **Tyr** | **Plant species** | | **References** | |  |  |  |  |  |  |
| --- | --- | --- | --- | --- | --- | --- | --- | --- | --- | --- | --- | --- | --- | --- |
| ***Stress-related proteins*** | |  | |  |  | |  | |  |  |  |  |  |  |
| Cyclin-dependent kinase A (CDKA)  NADP-malic enzyme 2  Thaumatin-like protein E2  Mitochondrial manganese superoxide dismutase (MSD1) | | Decreased activity  Decreased activity  Not defined  Inhibition | | Tyr-15, Tyr-19^(2)^  Tyr-73^(2)^  Tyr-36^(2)^  Tyr-63^(3)^ | *Zea mays*  *Arabidopsis thaliana*  *Nicotiana tabacum*  *Arabidopsis thaliana* | | Méndez et al., (2020)  Begara-Morales et al., (2019)  Takahashi et al. (2016)  Holzmeister et al., 2015 | |  |  |  |  |  |  |
| Monodehydro-ascorbate reductase (MDAR)  ABA receptor PYR1  ABA receptor PYL4  ABA receptor PYL8 | | Inhibition  Inhibition  Inhibition  Inhibition | | Tyr-345^(2)^  Tyr-58, Tyr-120 ^(2)^  Tyr-80^(2)^  Tyr-60, Tyr-158^(2)^ | *Pisum sativum*  *Arabidopsis thaliana*  *Arabidopsis thaliana*  *Arabidopsis thaliana* | | Begara-Morales et al., 2015  Castillo et al., (2015)  Castillo et al., (2015)  Castillo et al., (2015) | |  |  |  |  |  |  |
| Ascorbate peroxidase (APX)  Glutathione reductase (GR)  Glyceraldehyde-3-phosphate dehydrogenase (NADP-GAPDH) | | Decreased activity  No effect  Inhibition | | Tyr-235^(2)^  Tyr-23^(1)^  Tyr-318^(2)^ | *Pisum sativum*  *Helianthus annuus,*  *Pisum sativum*  *Arabidopsis thaliana* | | Begara-Morales et al., 2013  Chaki et al., 2009;  Begara-Morales et al., 2014  Lozano-Juste et al., (2011) | |  |  |  |  |  |  |
| ***Metabolic enzymes***  Nitrate reductase (NIA1)  Nitrate reductase (NIA2)  Nitrite reductase 1 (NiR1) | | Decreased activity  Decreased activity  Decreased activity | | Tyr-714^(2)^  Tyr-733^(2)^  Tyr-147, Tyr-155, Tyr-414, Tyr-553^(1)^ | *Arabidopsis*  *thaliana*  *Arabidopsis*  *thaliana*  *Arabidopsis*  *thaliana* | | Costa-Broseta et al., (2021)  Costa-Broseta et al., (2021)  Costa-Broseta et al., (2020) | |  |  |  |  |  |  |
| Leghemoglobin (Lb) | | Inactivation | | Tyr-30^(2)^ | *Phaseolus vulgaris*  nodules | | Sainz et al., 2015 | |  |  |  |  |  |  |
| NADP-isocitrate dehydrogenase | | Decreased activity | | Tyr-392^(2)^  Tyr450^(3)^ | *Pisum sativum*  *Arabidopsis. thaliana* | | Begara-Morales et al., 2013 | |  |  |  |  |  |  |
| Hydroxypyruvate reductase (HPR1) | | Decreased activity | | Tyr-198^(2,3)^ | *Pisum sativum* (peroxisomes)  *Arabidopsis thaliana* | | Corpas et al., 2013a  Corpas et al., 2013b | |  |  |  |  |  |  |
| Glutamine synthetase | | Enzyme inactivation | | Tyr-167^(2,3)^ | *Medicago tranculata* | | Melo et al., 2011 | |  |  |  |  |  |  |
| O-acetylserine(thiol) lyase A1 | | Decreased activity | | Tyr-302^(2)^ | *Arabidopsis thaliana* | | Alvarez et al., 2011 | |  |  |  |  |  |  |
| S-adenosyl homocysteine hydrolase (SAHH) | | Decreased activity | | Tyr-448^(1)^ | *Helianthus*  *annuus* | | Chaki et al., 2009 | |  |  |  |  |  |  |
| ***Proteins involved in photosynthetic processes*** | | |  |  | |  | |  |  |  |  |  |  |  |
| Photosystem II protein (PsbO1) | Unknown | | | Tyr-9^(2)^ | *Arabidopsis*  *thaliana* | | Takahashi et al., 2015 | |  |  |  |  |  |  |
| Carbonic anhydrase (β-CA) | Decreased activity | | | Tyr-205 ^(1)^ | *Helianthus*  *annuus* | | Chaki et al., 2013 | |  |  |  |  |  |  |
| PSBA(D1) of Photosystem II complex | Disassembly of PSII dimers | | | Tyr-262^(2)^ | *Arabidopsis*  *thaliana* | | Galetskiy et al., 2011 | |  |  |  |  |  |  |
| Methionine synthase | Decreased activity | | | Tyr-287^(2)^ | *Arabidopsis*  *Thaliana* | | Lozano-Juste et al., 2011 | |  |  |  |  |  |  |

^(1)^ In silico identification.

^(2)^ Mass spectrometric techniques (LC-MS/MS).

^(3)^ Site-Directed Mutagenesis

**Table S2.** Sequences of primers used for the RT-qPCR reaction.

| gene | NCBI reference sequence | forward primer | reverse primer |
| --- | --- | --- | --- |
| *EF1-α* | AB061263 | TCCTTACCTGAACGCCTGTCA | ATTGGAAACGGATATGCTCCA |
| *PiTef1* | AJ249839 | TCCACACACAAAGTGCATCA | ATGACTCGCCTCGGTGATTA |
| *PR2* | U01901 | TGTTGCCACCAACATTCACA | TCAATGTTTGATCCTCTTAACGCA |

**Table S3.** Characterization of all 15 tyrosine (Y) residues present in potato β-1,3-glucanase in terms of amino acid content within a nine-residue window of linear sequence around the tyrosine (Primary structure), solvent-accessible surface area (ASA), secondary-structure organization, and residues within 5 Å from the two carbons susceptible to nitration. Residues meeting the nearby-residue criterion (basic amino acids in the immediate vicinity of the tyrosine residue within the primary sequence and/or the acidic residues within 5 Å from the atom susceptible to nitration) are shown in bold (B).

| **Tyrosine residues (Y)** | **Conservation score (1-9)** | **Primary structure** | **ASA (Å^2^)** | **Secondary structure** | **Residues within 5 Å from the potential nitration target (atom)** |
| --- | --- | --- | --- | --- | --- |
| Y31 | 9 | LGVCY**^31^**GMMG | 3.8 | loop | Ile45, Val44, Leu58, Phe311, Gly32, Met33, His41, Ala65 |
| Y48 | 5 | VIQLY**^48^K**S**R**N | 0.0 | alpha helix | Leu56, Arg55, Ile53, Val44, Met310, Tyr31, Val29 |
| Y59 | 9 | **R**L**R**LY**^59^**DPNQ | 49 | loop | **Glu265**, Cys30, Tyr31, Arg57, Leu58, Asn119, Phe308, Gly32 |
| Y114 | 8 | V**K**I**K**Y**^114^**IAVG | 25.5 | beta sheet | Lys155, Pro197, Val262, **Glu304**, Lys113, Ile77, Tyr306, Leu199 |
| Y142 | 3 | LVNIY**^142^**KAVG | 83.4 | alpha helix | Lys143, Thr194 |
| Y169 | 3 | IGNSY**^169^**PPSQ | 156.9 | loop | Val229, Pro170 |
| Y203 | 9 | LVNIY**^203^**PYFS | 27.5 | loop | **Glu265**, Phe324, Gly267, Cys266, Asn201, Ile202, Phe206, Phe308 |
| Y205 | 9 | NIYPY**^205^**FSYS | 2.6 | alpha helix | Leu320, Pro269, Ala276, Phe274, Ser209, Phe206, Trp268, Gly275 |
| Y208 | 7 | PYFSY**^208^**SGNP | 24.3 | alpha helix | Phe274, Ser209, Pro212, Leu217, Ser216, Ile215 |
| Y219 | 9 | ISLPY**^219^**ALFT | 17.4 | alpha helix | Ile215, Leu239, Ala242, Asn238, Tyr236, Val229, Arg237, Val228 |
| Y236 | 9 | GS**R**QY**^236^R**NLF | 1.4 | beta sheet | Arg234, **Asp231**, Gln235, Pro171, Pro297, **Asp245**, Ala242, **Asp241** |
| Y248 | 7 | LDSVY**^248^**AAME | 22 | alpha helix | **Asp245**, Leu244, Ser296, Lys299, Ile261, Gly260, Val259 |
| Y284 | 9 | NAATY**^284^**L**R**NL | 0.0 | alpha helix | Cys266, Leu288, Gly267, Leu285, Ala220, Phe222, Pro204, Trp268 |
| Y306 | 9 | PIETY**^306^**IFAM | 0.0 | beta sheet | Val263, Thr305, Val29, Gly28, **Glu304**, Arg55, Ile77, Arg57, Leu56, Val262, Tyr114 |
| Y335 | 6 | KQPKY**^335^**NLNF | 24.1 | loop | Lys334, Leu326, Gly325, Ser270, Gln278, Ala281, Leu285, Ala282 |

Supplementary Figure 1. Validation of protein nitration. Commercial nitrated bovine serum albumin was used as a positive control to confirm the detection of nitrated residues in recombinant β-1,3-glucanase.


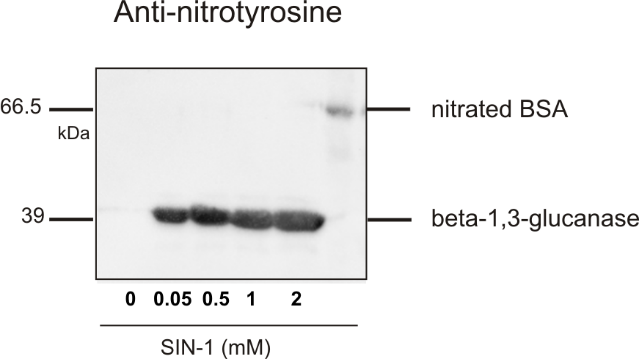

Supplement: Supplementary file 1 [file DataSheet1.docx]
